# Variation of phenotypic and physiological traits of *Robinia pseudoacacia* L. from 20 provenances

**DOI:** 10.1371/journal.pone.0262278

**Published:** 2022-01-05

**Authors:** Qi Guo, Yuhan Sun, Jiangtao Zhang, Yun Li

**Affiliations:** 1 College of Agriculture/Tree Peony, Henan University of Science and Technology, Luoyang, Henan, China; 2 Beijing Advanced Innovation Center for Tree Breeding by Molecular Design, Beijing Forestry University, Beijing, China; 3 National Engineering Laboratory for Tree Breeding, Beijing Forestry University, Beijing, China; 4 College of Biological Sciences and Technology, Beijing Forestry University, Beijing, China; 5 Henan Academy of Forestry, Zhengzhou, China; National Bureau of Plant Genetic Resources, INDIA

## Abstract

To select elite *Robinia pseudoacacia* L. germplasm resources for production, 13 phenotypes and three physiological indicators of 214 seedlings from 20 provenances were systematically evaluated and analyzed. The leaf phenotypic and physiological coefficients of variation among the genotypes ranged from 3.741% to 19.599% and from 8.260% to 42.363%, respectively. The Kentucky provenance had the largest coefficient of variation (18.541%). The average differentiation coefficients between and within provenances were 34.161% and 38.756%, respectively. These close percentages showed that *R*. *pseudoacacia* presented high genetic variation among and within provenances, which can be useful for assisted migration and breeding programs. Furthermore, based on the results of correlations, principal component analysis and cluster analysis, breeding improvements targeting *R*. *pseudoacacia’s* ornamental value, food value, and stress resistance of were performed. Forty and 30 excellent individuals, accounting for 18.692% and 14.019%, respectively, of the total resources. They were ultimately screened, after comprehensively taking into considering leaf phenotypic traits including compound leaf length, leaflet number and leaflet area and physiological characteristics including proline and soluble protein contents. These selected individuals could provide a base material for improved variety conservation and selection.

## Introduction

A germplasm resources is a collection of all the genes of a species’ individuals and play an important role in developing new varieties, discovering important agronomic traits, conserving endangered species, maintaining ecological balance and stabilizing the environment [1–3]. The germplasm can be considered as a carrier of biodiversity and is highly important [4]. *In situ* preservation and *ex situ* preservation are the two standard methods for the protection of germplasm resources. Although *in situ* conservation maintains the original ecosystem and natural habitat of plants better than *ex situ* preservation, it requires a large cultivation area and large investments of labor, materials, finances, and time for administration and management. *Ex situ* preservation acts as a backup for certain aspects of diversity that might otherwise be lost in human-dominant ecosystems and in nature [5–7]. Forest trees require a long period and large area for growth, and *ex situ* preservation is commonly used to protect plant resources. This method is convenient for breeders as it, allows research to be carried out in in a timely and efficient manner [8, 9].

Since the early 1990s, China began to carry out systematic research work on protecting forest genetic resources. A preservation system for forest genetic resources was established for country’s actual situation, by forming a framework for preserving and utilizing forest germplasm resources. It was coordinated by the National Forest Germplasm Resources Platform and National Forest Base. Breeding efforts have been carried out on many endangered species, such as *Taxus chinensis* var. Mairei [10, 11], dove tree (*Davidia involucrata* Baill) [12], *Emmenopterys henryi* Oliv. [13, 14], and *Cathaya argyrophylla* [15], so that populations of endangered species can expand. Forest germplasm resources with excellent characteristics and important economic value have been examined and approved for improved, new and local varieties. By this means, excellent seedlings and propagation materials should be produced by establishing seed standards, seed orchards, cutting orchards, demonstration forests, and so on. The goal of tree genetic resource preservation is to create ecological and social benefits to maintain the sustainable development of a biological environment. Nevertheless, the utilization of forest resources is still occurring at a slow pace, given the destruction of the environment and the demand for economic development. This situation can be reversed by paying attention to the investigation, protection and utilization of the forest germplasm; increasing funding support for forest resource research, improving the publicity and public education about forest genetic resources; and raising the whole population’s awareness to protect genetic resources [16–20].

Black locust (*Robinia pseudoacacia* L.) is a multipurpose deciduous tree species. It is suitable for land reclamation, windbreaks, fence posts, raw material for energy plantations, timber, bee-keeping, wood fibre, and forage [21, 22]. It was first introduced to China in 1877, and been extensively planted in 27 provinces [23, 24]. *Robinia pseudoacacia* L. has become an important pioneer afforestation tree in northwest China because of it can withstand slight saline-alkaline and dry soils and has grown well in barren mountains [25]. In addition, the black locust is an economically valuable tree: bees feed on its flowers to produce honey, the forest rapidly growths, and the durability and strength its timbers make it suitable for building [26–28]. However, the haphazard introduction of black locust into many areas of China and a related lack of records about introduced samples have led to confusion concerning the black locust germplasm resources in China. This has greatly restricted breeding efforts and effective utilization of the black locust. The germplasm resources of black locust were systematically utilized and protected until the target-oriented breeding stage began in the 1990s [29–32].

A prerequisite for plant breeding is the research of genetic variation among individuals and groups of individuals. Plant phenotypes and physiological traits are affected by environmental conditions. Therefore, different plant phenotypes and physiological indicators can reflect the degree of plant adaptability to current site conditions [33, 34]. Knowledge about trait variation can provide a more comprehensive understanding of germplasm resource diversity among breeding materials. It can be used to carry out further targeted breeding work based on the characteristics of each germplasm resource. The degree of variation and pattern of germplasm resources can be determined relatively easily. This, overall concept forms the basis of genetic breeding and is the most common and effective method for breeders [35–37].

Unfortunately, there have been few studies of the differences in the provenance of leaf phenotypic and physiological parameters of various black locust collected within their natural distribution [38]. However, an analysis based on morphological characteristics including germination ability, plant height and diameter at the ground level was performed to evaluated the seed characteristics and variation of 19 black locust from provenances in China. The results showed that interactions between the genotype and environment caused significant differences in tree growth from those provenances under different site conditions [39]. Zhang et al. [40] analyzed the seed survival rate, average height and diameter at breast height (DBH) of black locust trees from different Chinese provenances. They found that the further the provenance was from the test site, the lower its the survival and growth rates. At the origin provenance level, Zhou [41] investigated the differences in fruits, seeds, seedling height and diameter at the ground level for annual seedlings from different original black locust provenances. He also assessed the geographical variation in traits of different provenances, and made a preliminary selection of two provenances with high growth. Li et al. [42] surveyed 183 black locust families from 38 provenances and found that the regular geographic variation reflected in the origin of black locust’s cold resistance presented a meridional gradient, which increased with increasing longitude.

To explore the diversity of leaf phenotypes and physiology based on natural black locust areas, 20 black locust provenances, 19 provenances covering almost the entire natural distribution and one provenance from China (CN), were used to comparatively analyze the variation among sixteen measured traits. Following that some elite trees were selected. The results provide valuable resources for efficient breeding and germplasm preservation of black locust trees in the future.

## Materials and methods

### Plant materials

In this study, 214 samples of black locust were collected from 20 locations from September to October 2010. They comprised the 19 main black locust natural distribution areas in the United States and one main cultivation area in Henan, China (Table 1) [43]. In these areas, several fruits of black locusts with normal growth and a diameter at breast height (DBH) greater than 20 cm were collected at 500 m intervals and mailed to the Henan Academy of Forestry Sciences in China. From April to July 2011, these collected seeds were placed in a greenhouse for 24 h until germination. The successfully germinated seedlings were placed in nutrient bowls filled with nutritive soil and managed normally until the seedlings’ height reached approximately 30 cm high, and were then transplanted to Mengjin Forest Farm (34°49′18″N, 112°28′12″E), Luoyang, Henan, China. Mengjin County is a transition zone between subtropical and temperate; the annual average temperature was 15.4°C, and the annual average precipitation was 593 mm from 2010 to 2017, respectively. The soil is mainly brown soil (accounting for 93%), followed by alluvial soil (accounting for 7%). There was at least one site for each provenance, and each site contained at least two accessions randomly distributed with a plant spacing of 4 m×4 m.

**Table 1:** Information on the 20 *R*. *pseudoacacia* L. provenances.

Provenances Name	Sites	Accessions Numbers	Longitude	Latitude	Altitude (m)
VA (Virginia)	Blacksburg	6	37°26’	80°45’	646
	Independence	6	36°37’	81°07’	770
	Washington, D.C.	7	38°28’	76°3’	26
WV (Washington)	New River Gorge	7	38°04	81°03’	539
	Morgantown	6	39°37’	79°57’	322
	West Huntington	7	38°23’	82°29’	214
NC (North Carolina)	Huntersville	6	35°25’	80°51’	778
GA (Georgia)	Blue Ridge Lake	7	34°51’	84°19’	5915
MD (Maryland)	Old National Pike	5	39°42’	78°2’	2917
PA (Pennsylvania)	Big Beaver Blvd	7	40°33’	80°16’	347
	Bedford	7	40°07’	78°3’	390
OH (Ohio)	Cadiz Piedmont	6	40°1’	81°16’	310
	Cincinnati	5	39°03’	84°31’	216
IN (Indiana)	Fisher	6	39°56’	85°54’	259
	Georgetown	7	38°17’	85°55’	260
	Elberfeld	4	38°1’	87°26’	129
IL (Illinois)	Bloomington	6	40°28’	89°01’	235
	Toe Exit	6	39°03’	88°4’	202
KY (Kentucky)	Mt. sterling	6	38°03’	84°02’	306
	Bowling Green	7	37°	86°17’	178
	Kentucky Lake	4	38°30’	85°57’	306
	Wickliffe	5	37°01’	89°03’	113
TN (Tennessee)	Knoxville	7	35°52’	83°57’	258
	Waverly	7	35°53’	87°39’	154
MS (Mississippi)	Sardis	4	34°26’	89°53’	101
AL (Alabama)	Upper Elkton Rd	6	34°56’	86°53’	258
MS/AL (Mississippi/Alabama)	MS/AL border	6	34°11’	88°06’	188
IA (Iowa)	Burlington	5	40°5’	91°08’	194
MO (Missouri)	Hannibal	5	39°43’	91°21’	178
	St. James	7	38°	91°31’	278
KS (Kansas)	Riverton	2	37°06’	94°42’	247
OK (Oklahoma)	Pryor	6	36°19’	95°18’	183
AR (Arkansas)	Colt	3	35°06’	90°46’	114
CN	China	23	112°32’	34°80’	243
Total	-	214	-	-	-

Notes: VA, Virginia; WV, Washington; NC, North Carolina; GA, Georgia; MD, Maryland; PA, Pennsylvania; OH, Ohio; IN, Indiana; IL, Illinois; KY, Kentucky; TN, Tennessee; MS, Mississippi; AL, Alabama; IA, Iowa; MO, Missouri; KS, Kansas; OK, Oklahoma; AR, Arkansas.

Two hundred and fourteen well-grown black locusts of different provenances were selected as experimental material from the Mengjin Forest Farm in August 2017. Each specimen was chosen by selecting those whose annual branches were free of pests and disease at the same height in four directions (north, south, east, and west).

### Data collection

In this study, 16 quantitative traits of black locust trees were evaluated. They included 13 leaf traits: compound leaf length (CLL), compound leaf width (CLW), compound leaf length/width (CLL/CLW), compound petiole length (CPL), leaflet length (LL), leaflet width (LW), leaflet length/width (LL/LW), leaflet area (LA), leaflet perimeter (LPM), leaflet circularity (LC), leaflet pairs (LP), leaflet number (LN), and petiole angle (PA). The 16 traits also included three physiological traits: chlorophyll content (Chl), total protein content (Spro), and proline (PRO). The 13 leaf traits and Chl were evaluated at maturity in August 2017. The two remaining physiological traits were measured in healthy leaves from April to May 2017 (Table 2). The leaves were collected from trees and rapidly placed in sealed bags containing dry ice, and then transferred to the National Engineering Laboratory for Tree Breeding, Beijing Forestry University, China (40°0′22″N, 116°21′1″E), and stored at -80°C until tested. The compound leaf traits were measured by a ruler with a precision of 0.01 cm. The petiole angle was surveyed by an electronic protractor with a precision of 0.01°. The leaves were scanned and saved in the same manner, and the remaining leaf characteristics were analyzed using LAMINA version 1.0.2 software. The chlorophyll content in mature leaves of black locust was determined by a SPAD-502 Plus chlorophyll meter (Konica Minolta, Japan); three leaflets were selected randomly from each direction (north, south, east, and west), and each leaflet was measured at 6 positions. Following that, the results were averaged into one measurement. Total protein content and proline were evaluated with a Total Protein Assay Kit (Art. No. A045-4) and a Proline Assay Kit (Art. No. A107), produced by the Nanjing Jiancheng Bioengineering Institute (http://www.njjcbio.com/).

**Table 2:** Investigated information of *R*. *pseudoacacia* L. germplasm.

Traits	Abbreviation
Compound Leaf Length	CLL
Compound Leaf Width	CLW
Compound Leaf Length/Width	CLL/CLW
Compound Petiole Length	CPL
Leaflet Length	LL
Leaflet Width	LW
Leaflet Length/Width	LL/LW
Leaflet Area	LA
Leaflet Perimeter	LPM
Leaflet Circularity	LC
Leaflet Pairs	LP
Leaflet Numbers	LN
Petiole Angle	PA
Chlorophyll Content-SPAD value	Chl
Soluble Protein Content	Spro
Proline Content	PRO

### Data and statistics

Microsoft Excel 2016 was used to examine the variation in leaf phenotypic and physiological traits, including the mean value, standard error, amplitude, and coefficient of variation (*CV*). SPSS version 24 was used to perform analyses of variance (ANOVAs) in conjunction with Duncan’s multiple range tests for multiple comparisons. Principal component analysis was applied to the sixteen traits of the black locust provenances. A p-value for the ANOVA *F* tests ≤0.05 was considered significant. The formula, V*st* (%) = δ^2^*t/s*/(δ^2^*t/s*+δ^2^*s*), was used to calculate the percentage of variance among and within the provenances, where *Vst* is the differentiation coefficient of the trait, *δ*^*2*^_*t/s*_ is the variance component between provenances, and *δ*^*2*^_*s*_ is the variance component within provenances [44]. A covariance correlation matrix was then used to analyze the correlations between clonal populations and geographical populations. The euclidean distance of each quantitative trait was calculated with the open-source statistical package, R; graphical visualization of the results was carried out using MEGA ver. 6.0 [45] after all the tested data had been processed in SPSS version 24. In addition, Mantel’s correlation tests were conducted on the euclidean and geographical distances of all the traits of the black locust trees.

## Results

### Analysis of leaf phenotypic characteristics and physiological indicators

Table 3 shows the mean values and the multiple comparison results based on Duncan’s test. Compound leaves and leaflets differed between the 20 provenances. Between the four compound leaf traits, there was no significant difference in compound leaf length (CLL), and the length ranged from 26.426 cm (CN) to 29.710 cm (IL). Compared with those at the other locations, the accessions in MS, with the largest value of 2.916 cm, showed a significant difference in CLL/CLW traits. Of the nine leaflet characteristics, there were no significant differences in petiole angle (PA) between accessions in LA. The accessions in MS had the smallest LL and LP, whereas those in KS had the largest LP and LN, with averages of 9.971 and 20.833, respectively. Similarly, the results for the multiple comparison tests of the three physiological traits showed no significant difference in proline content between provenances, but both the chlorophyll content and soluble protein content had significant differences. Among the above indicators, the provenances in IN, KS, and OH showed the highest values of certain characteristics—38.22±0.69 (Chl-SPAD value), 1445.861±899.893 μg·ml^-1^ (Spro) and 44.649±11.401 μg·g^-1^ (PRO), respectively. However, the provenances in KS, AR, and OK had the lowest values—31.98±0.54 (Chl-SPAD value), 1445.861±899.893 μg·ml^-1^ (Spro), and 19.257±3.532 μg·g^-1^ (PRO), respectively.

**Table 3:** Differences in leaf phenotypic traits of 20 *R*. *pseudoacacia* L. provenances.

Provenance	Sample No.	CLL (cm)	CLW (cm)	CLL/CLW	CPL (cm)	LL (cm)	LW (cm)	LL/LW	LA (cm^2^)	LPM (cm)	LC (%)	LP (pair)	LN (piece)	PA (°)	Chl (SPAD value)	Spro (μg·ml^-1^)	PRO (μg·g^-1^)
		Average±SE	Average±SE	Average±SE	Average±SE	Average±SE	Average±SE	Average±SE	Average±SE	Average±SE	Average±SE	Average±SE	Average±SE	Average±SE	Average±SE	Average±SE	Average±SE
VA	19	28.41±0.42	11.94±0.36b	2.43±0.07ab	3.82±0.08ab	7.178±0.207ab	3.065±0.078bc	2.357±0.056a	15.967±0.792bc	15.755±0.432ab	76.367±0.870b	7.62±0.16abcd	16.23±0.32abcd	56.25±3.30	35.33±0.80abcd	954.628±99.265ab	33.079±4.836
WV	20	27.04±0.64	11.10±0.34ab	2.47±0.05ab	3.55±0.08ab	6.843±0.172ab	2.891±0.064bc	2.383±0.052a	14.388±0.661abc	15.038±0.402ab	76.274±0.849b	7.92±0.20abcdef	16.74±0.39abcdef	50.77±2.07	37.06±0.79cd	971.115±103.529ab	40.836±7.789
NC	6	28.47±1.07	11.34±0.44ab	2.56±0.15ab	4.06±0.15b	7.134±0.193ab	3.240±0.061c	2.208±0.056a	16.661±0.580c	15.930±0.434ab	78.166±1.082b	7.44±0.44abc	15.88±0.88abc	56.94±2.34	36.59±2.24cd	724.562±29.806a	22.042±2.021
GA	7	28.37±1.06	11.95±0.55b	2.40±0.09ab	3.78±0.13ab	7.066±0.348ab	3.169±0.168bc	2.246±0.055a	16.293±1.600c	15.961±0.906ab	77.448±1.057b	7.29±0.31ab	15.50±0.61ab	60.74±2.90	32.49±0.81ab	1062.785±233.458ab	26.043±2.657
MD	5	27.55±1.10	10.77±0.81ab	2.61±0.16abc	3.84±0.33ab	6.656±0.386ab	2.979±0.147bc	2.239±0.042a	14.436±1.425abc	14.807±0.839ab	77.950±0.545b	7.67±0.33abcd	16.27±0.66abcd	49.95±4.32	37.80±1.40cd	575.798±82.213a	25.411±2.145
PA	14	29.09±1.21	11.30±0.50ab	2.61±0.07abc	3.94±0.16b	7.042±0.264ab	3.046±0.101bc	2.321±0.049a	15.607±1.045bc	15.431±0.525ab	76.623±0.688b	7.80±0.23abcdef	16.56±0.47abcdef	56.31±2.74	36.46±0.60bcd	752.702±100.970a	25.159±1.999
OH	11	27.53±0.94	10.94±0.43ab	2.56±0.07ab	3.61±0.16ab	6.849±0.151ab	2.948±0.076bc	2.340±0.052a	14.544±0.630abc	14.951±0.338ab	76.425±0.792b	7.70±0.21abcde	16.39±0.42abcde	54.62±3.35	36.01±0.86abcd	1053.431±154.133	44.649±11.401
IN	17	28.28±0.75	11.56±0.30ab	2.47±0.07ab	3.72±0.11ab	6.976±0.146ab	2.997±0.085bc	2.352±0.054a	15.126±0.691abc	15.384±0.368ab	76.356±0.811b	8.26±0.16bcdef	17.46±0.32bcdef	59.52±3.25	38.22±0.69d	1078.238±124.553	38.455±4.143
IL	12	29.71±0.90	10.86±0.40ab	2.76±0.10bc	3.81±0.11ab	6.849±0.291ab	2.916±0.069bc	2.362±0.087a	14.392±0.858abc	15.148±0.720ab	76.036±1.086b	8.84±0.28f	18.59±0.56f	57.81±2.17	38.18±0.88d	880.659±114.138a	30.843±3.053
KY	22	27.35±0.64	11.20±0.29ab	2.489±0.073ab	3.65±0.08ab	6.910±0.120ab	2.997±0.072bc	2.323±0.041a	14.914±0.575abc	15.101±0.269ab	76.317±0.624b	7.36±0.19ab	15.66±0.37ab	63.27±1.27	35.99±0.77abcd	1015.229±97.650ab	41.605±9.150
TN	14	26.54±1.11	10.96±0.45ab	2.448±0.066ab	3.79±0.16ab	6.614±0.293ab	2.932±0.113bc	2.273±0.077a	14.122±1.123abc	14.713±0.554ab	77.007±1.241a	7.09±0.17a	15.10±0.34a	57.96±3.83	33.97±0.75abc	745.232±85.245a	26.633±2.163
MS	4	27.87±1.83	9.85±0.78a	2.916±0.333c	3.33±0.15a	6.253±0.291a	2.749±0.177b	2.311±0.199a	12.247±0.982ab	13.890±0.371a	76.223±2.808b	8.77±0.94ef	18.48±1.85ef	55.60±6.01	34.11±2.17abcd	937.617±67.569ab	21.164±1.561
AL	6	28.98±1.04	11.29±0.29ab	2.580±0.080abc	3.83±0.23ab	6.804±0.223ab	3.105±0.101bc	2.201±0.040a	15.307±0.861abc	15.214±0.536ab	78.666±0.771b	8.58±0.48def	18.08±0.94def	62.69±2.42	36.65±1.08cd	817.918±155.467a	29.439±4.851
MS/AL	6	27.99±0.51	11.84±0.29b	2.381±0.048a	3.99±0.15b	7.073±0.153ab	2.956±0.091bc	2.408±0.076a	14.981±0.601abc	15.467±0.362ab	75.374±0.937b	8.17±0.33bcdef	17.31±0.65bcdef	57.95±2.89	35.25±1.11abcd	687.342±86.519a	26.051±3.814
IA	5	26.97±1.11	10.42±0.53ab	2.612±0.089	3.32±0.06a	6.653±0.430ab	2.809±0.133b	2.375±0.091a	13.594±1.484abc	14.979±0.975ab	76.327±1.427b	8.50±0.46cdef	17.90±0.87cdef	58.59±4.55	37.55±1.40cd	573.537±79.940a	42.153±8.507
MO	12	28.37±0.78	11.22±0.34ab	2.570±0.078abc	4.09±0.13b	6.891±0.239ab	3.141±0.079bc	2.202±0.051a	15.893±0.886bc	15.386±0.523ab	79.131±0.941b	7.94±0.27abcdef	16.84±0.55abcdef	56.41±2.40	36.62±0.74cd	728.183±78.173a	30.918±3.378
KS	2	29.71±2.79	11.13±1.30ab	2.689±0.055abc	3.51±0.13ab	6.607±0.729ab	2.380±0.241a	2.783±0.026b	11.559±2.448a	14.057±1.515a	71.565±0.116a	9.92±0.08g	20.83±0.17g	64.19±12.08	31.98±0.54a	1445.861±899.893b	31.065±1.182
OK	6	28.64±1.34	11.10±0.37ab	2.607±0.113abc	3.95±0.20b	6.871±0.267ab	3.177±0.157bc	2.188±0.095a	15.617±1.244bc	15.196±0.611ab	77.754±1.253b	8.08±0.23abcdef	17.08±0.45abcdef	62.91±2.32	32.50±1.45ab	829.219±89.466a	19.257±3.532
AR	3	28.28±2.25	11.61±0.98ab	2.464±0.139ab	3.54±0.19ab	7.343±0.366b	3.165±0.151bc	2.335±0.084a	16.586±1.511c	16.508±0.532b	75.594±0.242b	7.86±0.35abcdef	16.67±0.67abcdef	57.18±6.27	34.70±0.67abcd	560.188±168.537a	20.291±3.339
CN	23	26.43±0.60	11.31±0.29ab	2.365±0.052a	3.59±0.10ab	6.758±0.142ab	2.975±0.067bc	2.288±0.045a	14.499±0.542abc	14.866±0.336ab	76.942±0.720b	7.27±0.15ab	15.53±0.31ab	59.27±2.77	35.91±0.63abcd	978.708±98.483ab	28.244±2.964
Total	214	28.09±1.10	11.19±0.50	2.550±0.098	3.74±0.14	6.869±0.271	2.982±0.112	2.325±0.066	14.837±1.027	15.189±0.577	76.627±0.943	8.00±0.30	16.95±0.59	57.95±3.66	35.67±1.02	868.648±147.45	30.167±4.224
Mean square		10.182	1.763	0.147	0.370	0.388	0.151	0.063	8.815	2.013	11.223	3.12	12.10	144.013	25.507	268823.008	586.728
*F*-value		1.087	0.866	1.802	1.786	0.635	1.426	1.312	0.948	0.673	1.000	4.34	4.31	1.252	2.554	1.494	1.127
*p*-value		0.366	0.625	0.025	0.027	0.876	0.118	0.179	0.524	0.843	0.462	0.00	0.00	0.220	0.001	0.091	0.327

Note

^a^ The first homogeneous group at 95% confidence for each trait (Duncan’s multiple range test).

^b^ The second homogeneous group at 95% confidence for each trait (Duncan’s multiple range test).

^c^ The third homogeneous group at 95% confidence for each trait (Duncan’s multiple range test).

^d^ The fourth homogeneous group at 95% confidence for each trait (Duncan’s multiple range test).

^e^ The fifth homogeneous group at 95% confidence for each trait (Duncan’s multiple range test).

^f^ The sixth homogeneous group at 95% confidence for each trait (Duncan’s multiple range test).

### Differentiation coefficient analysis of sixteen trait parameters

**Fig 1:**
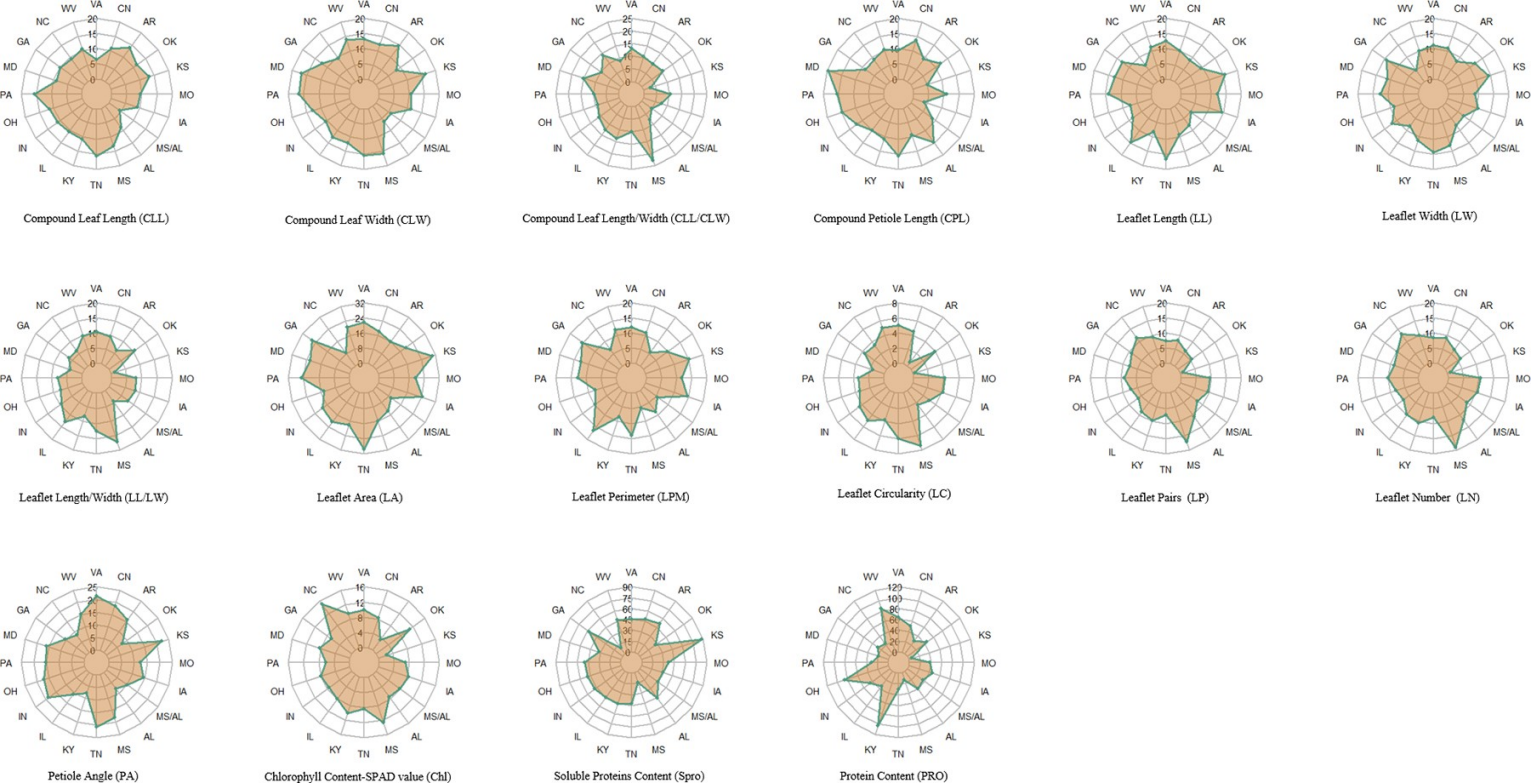
Distribution patterns of coefficients of variation of 16 tested traits of *R*. *pseudoacacia* L. in 20 provenances.

The analysis results (S1 Table in S1 File, Fig 1) showed that the coefficient of variation in the 13 leaf phenotypic traits among the different provenances was 3.741%-19.599%. All traits were lowest in LC and highest in LA, which indicates that LC has the smallest dispersion degree and highest stability among the leaf phenotypic traits; the case of LA is completely opposite. The coefficient of variation of the compound leaf traits was 11.236% and close to that of the leaflet traits (11.301%). At the provenance level, the total coefficient of variation was 11.281%, with the lowest and highest coefficients of variation at 7.286% (MS/AL) and 14.788% (TN), respectively, which shows that the provenance in TN had the most abundant leaf phenotypic diversity among the 20 black locust provenances.

Similarly, the total average variation of the three physiological parameters was 30.993%, and the value of soluble proteins (Spro) (42.363%) had the highest level, followed by the proline (PRO) (42.356%) and chlorophyll content (Chl) (8.260%); Spro had the highest stability of these parameters, as revealed by the maximum-to-minimum ratios of each indicator—6.364%, 8.735% and 19.178%, respectively. Of the 20 provenances, KY provenance had the highest total average coefficient of variation (52.786%), which was approximately 3.8 times that for the MS provenance, which presented the smallest variation coefficient.

In conclusion, the variation richness of the 16 test indexes of the 214 black locust accessions was as follows: Spro (42.363%)>PRO (42.356%)>LA (19.599%)>PA (17.317%)>CLW (12.359%)>CPL (11.179%)>LL (10.942%)>LPM (10.741%)>CLL (10.708%)>CLL/CLW (10.697%)>LP (10.558%)>LW (10.522%)>LN (9.832%)>LL/LW (8.462%)>Chl (8.260%)>LC (3.741%). Similarly, the variation in richness at each provenance level was as follows: KY (18.514%)>WV (18.411%)>VA (17.323%)>OH (17.222%)>TN (17.105%)>CN (16.231%)>PA (16.174%)>IN (15.317%) = IL (15.317%)>GA (15.275%)>KS (14.943%)>MS (14.632%)>IA (14.606%)>MO (14.098%)>MD (13.852%)>OK (13.304%)>AL (13.298%)>AR (13.127%)>MS/AL (10.571%)>NC (10.224%).

### Analysis of the differentiation coefficients of sixteen trait parameters

The differentiation coefficient and variance components among/within provenances ranged from 25.843% (CLW) to 71.655% (PA) at the leaf phenotypic trait level, with an average of 48.829%. The mean leaf phenotypic differentiation coefficient of the nine leaflet traits was 52.259%, lower than that of four compound leaf indicators (41.112%). Thus the differentiation level for the leaflets was slightly higher than for the compound leaves. In terms of single traits, the differentiation coefficients of CPL, LW, LP, LN and PA were larger than those within provenances, indicating that the variation among provenances was higher than within them (Table 4). Furthermore, the total mean leaf phenotypic differentiation coefficient among provenances was lower than within provenances, suggesting that the main variation of black locust occurred intra-provenance, not inter-provenance variation.

**Table 4:** Variance percentages and differentiation coefficients of 16 traits among/within provenances of *R*. *pseudoacacia* L.

Traits	Variance component			Percentage of variance component			Differentiation coefficient (%)
	Among provenances	Within provenances	Random errors	Among provenances	Within provenances	Random errors	
Compound Leaf Length (CLL)	10.066	10.193	9.346	34.001	34.430	31.569	49.687
Compound Leaf Width (CLW)	1.733	4.973	1.974	19.965	57.293	22.742	25.843
Compound Leaf Length/Width (CLL/CLW)	0.101	0.203	0.079	26.371	53.003	20.627	33.224
Compound Petiole Length (CPL)	0.308	0.245	0.206	40.580	32.279	27.141	55.696
Leaflet Length (LL)	0.425	0.950	0.603	21.486	48.028	30.485	30.909
Leaflet Width (LW)	0.158	0.092	0.106	44.382	25.843	29.775	63.200
Leaflet Length/Width (LL/LW)	0.061	0.070	0.047	34.270	39.326	26.404	46.565
Leaflet Area (LA)	9.441	9.499	9.289	33.444	33.650	32.906	49.847
Leaflet Perimeter (LPM)	2.234	3.520	2.982	25.572	40.293	34.135	38.825
Leaflet Circularity (LC)	10.952	22.066	10.989	24.887	50.142	24.971	33.170
Leaflet Pairs (LP)	2.844	1.177	0.730	59.861	24.774	15.365	70.729
Leaflet Number (LN)	5.028	2.656	2.850	47.731	25.214	27.055	65.435
Petiole Angle (PA)	121.255	47.965	116.481	42.441	16.789	40.770	71.655
Chlorophyll Content-SPAD Value (Chl)	22.558	24.383	10.147	39.514	42.711	17.774	48.056
Soluble Protein Content (Spro)	200010.055	634891.966	170351.793	19.896	63.157	16.946	23.956
Proline Content (PRO)	485.974	503.046	521.181	32.179	33.310	34.511	49.137
Mean	-	-	-	34.161	38.756	27.074	47.246

Homoplastically, the variation of the three physiological parameters showed that Spro had the lowest and PRO, the highest differentiation coefficient at—23.956% and 49.137%, respectively. The total mean differentiation coefficient of the three indicators was 40.383%, but it was 59.617% within provenances. These results are consistent with the findings of leaf phenotypic traits, further demonstrating the importance of individual variation in black locust trees.

### Correlation analysis of sixteen trait parameters

**Fig 2:**
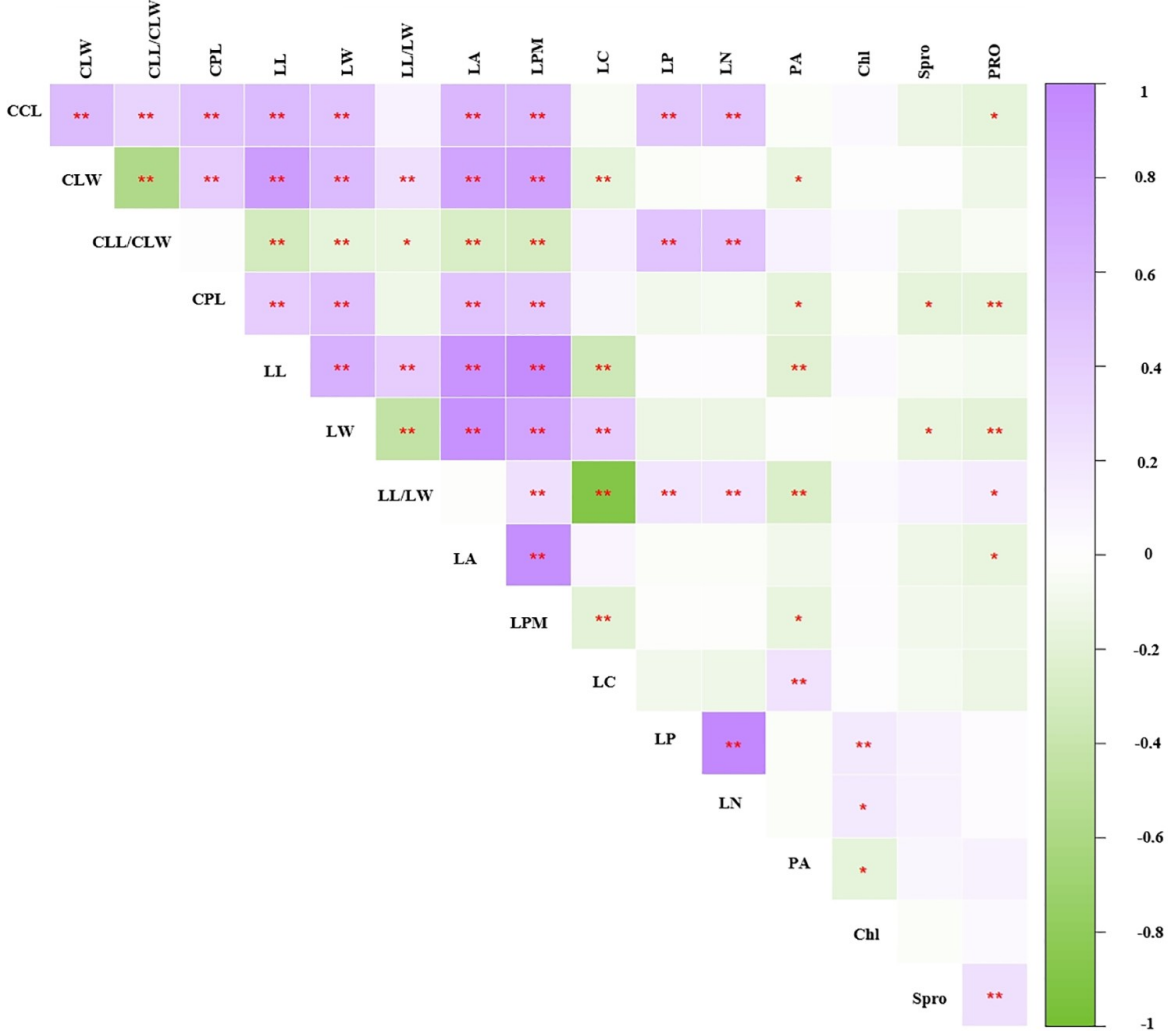
Heat map of a correlation analysis of 16 parameters from 214 *R*. *pseudoacacia* L. samples. Notes: *, P<0.05; **, P<0.01.

Sixty-one (50.833%) significantly correlated traits were identified by Pearson correlation analyses of the 16 parameters (CLL, CLW, CLL/CLW, CPL, LL, LW, LL/LW, LA, LPM, LC, LP, LN, PA, Chl, Spro, and PRO) in 20 *R*. *pseudoacacia* provenances (P<0.05 or P<0.01) (S2 Table in S1 File, Fig 2). Among these traits, there were 50 significantly correlated leaf phenotypic traits (P<0.05). For compound leaf traits, CLL/CLW showed a highly significant negative correlation with CLW (P<0.01) but was not significantly correlated with CPL (P>0.05). In terms of leaflets, LC showed significant negative correlations with LW, LL/LW, and LPM, and LW was significantly positively correlated with LA, LPM, and LC (P<0.01). However, CLL/CLW showed significant negative correlations with LL, LW, LA, LPM, and LP (P<0.01). There were 10 significantly correlations between phenotypic and physiological traits. CPL and LW exhibited extremely significant negative correlations between all pairs of physiological traits; Chl presented significant positive correlations with LP and LN, and PRO was significantly negatively correlated with CLW and LA (P<0.05). There were no significant correlations between physiological traits, except for a correlation between Spro and PRO.

### Principal component analysis of sixteen trait parameters

Five principal components explained the investigated characteristics with eigenvalues greater than 1.0 (Table 5), and the cumulative contribution rate reached 80.004%, essentially reflecting the main information contained in all the measured indexes. In principal component 1, the CLL, CLW, CPL, LL, LW, LA, and LPM with relatively high absolute values and were representative of some compound leaf and leaflet characteristics; in principal component 2, LP and LN were relative; CLL/CLW, LL/LW and LC constituted the principal component 3; and the principal component 4 emphasized PA, Spro, and PRO. The fifth component separated Chl.

**Table 5:** Principal component analysis of 16 traits of different *R*. *pseudoacacia* L. geographic provenances.

Tested traits	Eigenvector of the principal component				
	PC1	PC2	PC3	PC4	PC5
Compound Leaf Length (CLL)	0.680	0.441	0.412	-0.033	-0.160
Compound Leaf Width (CLW)	0.869	-0.002	-0.223	0.130	0.025
Compound Leaf Length/Width (CLL/CLW)	-0.305	0.458	0.631	-0.193	-0.171
Compound Petiole Length (CPL)	0.590	-0.113	0.248	-0.260	-0.051
Leaflet Length (LL)	0.940	0.138	-0.222	0.049	-0.030
Leaflet Width (LW)	0.802	-0.362	0.390	0.144	0.078
Leaflet Length/Width (LL/LW)	0.162	0.605	-0.722	-0.118	-0.137
Leaflet Area (LA)	0.954	-0.121	0.118	0.124	0.036
Leaflet Perimeter (LPM)	0.949	0.025	-0.100	0.067	-0.021
Leaflet Circularity (LC)	-0.107	-0.520	0.733	0.189	0.205
Leaflet Pairs (LP)	0.002	0.867	0.381	0.142	0.043
Leaflet Number (LN)	0.010	0.867	0.382	0.137	0.037
Petiole Angle (PA)	-0.194	-0.168	0.269	0.524	-0.409
Chlorophyll Content-SPAD value (Chl)	0.037	0.225	0.060	-0.113	0.854
Soluble Protein Content (Spro)	-0.133	0.152	-0.210	0.682	0.101
Proline Content (PRO)	-0.190	0.126	-0.263	0.559	0.194
Eigenvalue	5.124	2.839	2.442	1.320	1.075
Variance contribution rate (%)	32.027	17.743	15.263	8.249	6.722
Cumulative contribution rate (%)	32.027	49.771	65.033	73.282	80.004

### Cluster analysis based on sixteen trait parameters

**Fig 3:**
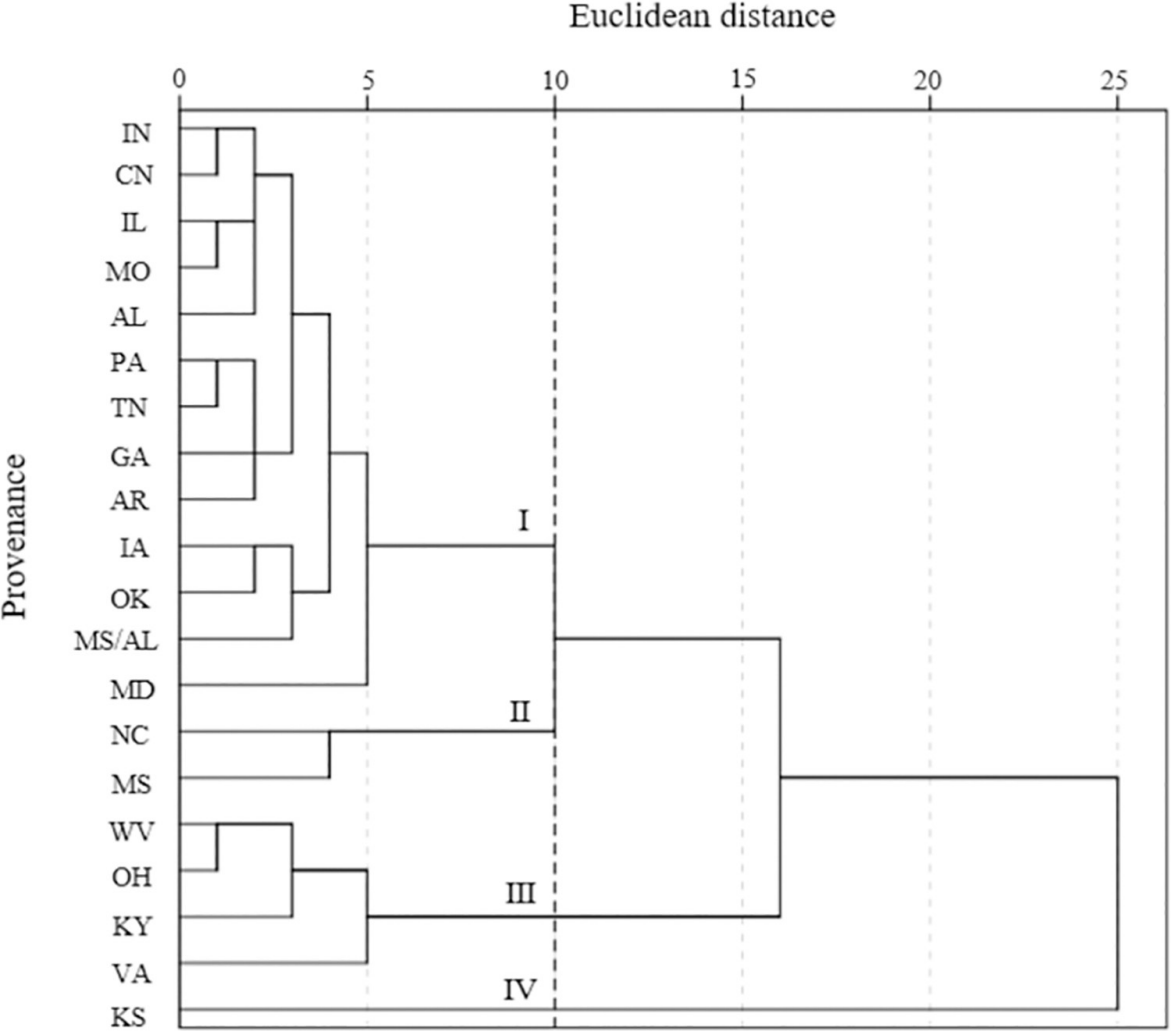
Cluster analysis based on leaf phenotypic and physiological traits of 20 *R*. *pseudoacacia* L. provenances.

Cluster analysis was performed for twenty different *R*. *pseudoacacia* provenances using the method of hierarchical cluster analysis between groups, taking sixteen trait parameters as variables (Fig 3). When the euclidean distance was set to 10, the twenty *R*. *pseudoacacia* provenances could be divided into four groups. Group 1 included 13 provenances (IN, CN, IL, MO, AL, PA, TN, GA, AR, IA, OK, MS/Al and MD), which had the largest CPL (3.875 cm), LW (3.028 cm), LA (15.112 cm^2^) and LPM (15.312 cm). Group 2 included the NC and MS provenances, which had the highest CLL/CLW (2.736 cm) and LC (77.194%). Group 3 contained WV, OH, KY and VA provenances, with the highest CLW (11.293 cm), LL (6.945 cm), Chl (36.096 SPAD) and PRO (40.042 μg·g^-1^). Group 4 comprised only one provenance, KS, which presented the highest CLL (29.709 cm), LL/LW (2.783), LP (9.917), LN (20.834), PA (64.192°) and Spro (1445.861 μg·ml^-1^).

**Fig 4:**
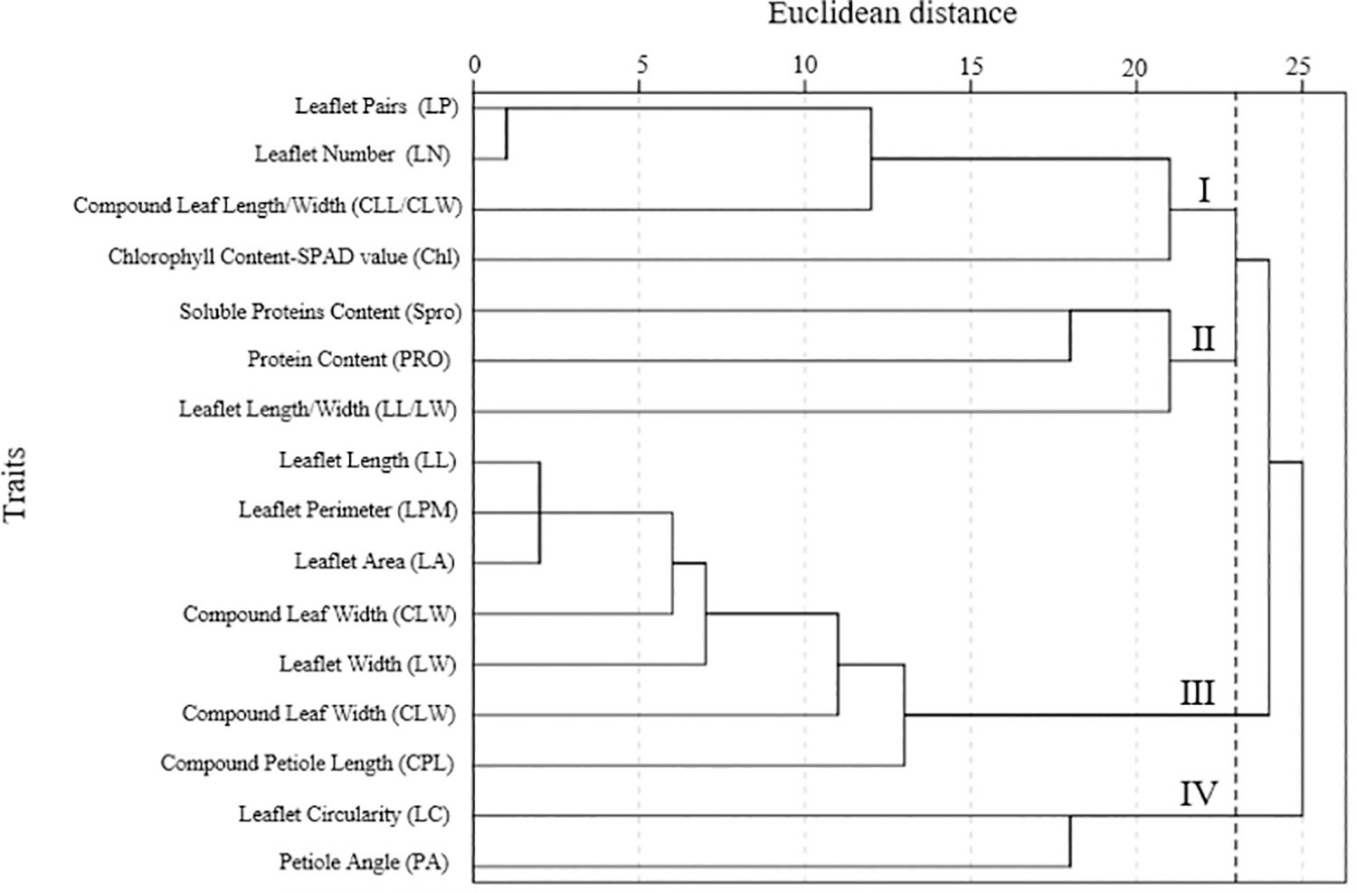
Cluster diagram of 16 test indicators of 214 samples.

In addition, based on the results of the principal component analysis, systematic clustering analysis was performed on the eigenvectors of all 16 parameters in the four principal components using the same method described above (Fig 4). The results showed that the sixteen indicators could be classified into four groups when the euclidean distance was set to 23: (1) LP, LN, CLL/CLW and Chl; (2) Spro, PRO and LL/LW; (3) LL, LPM, LA, CLW, LW and CLL; and (4) LC and LA.

### Mantel’s test based on sixteen trait parameters

**Fig 5:**
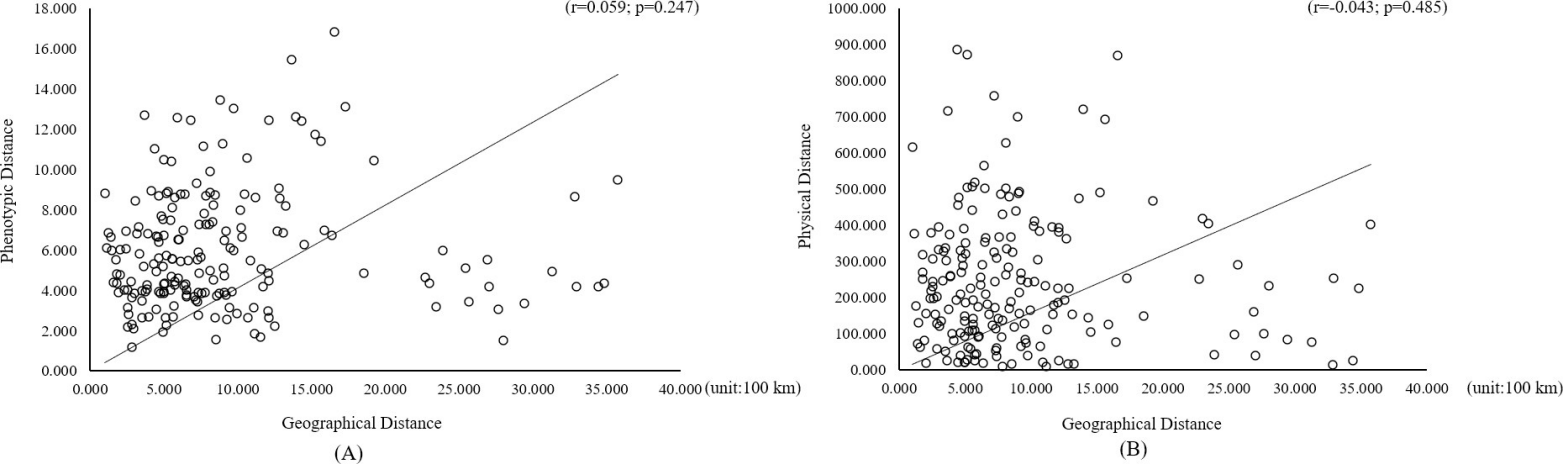
Relationships between geographical distance and tested trait distance for *R*. *pseudoacacia* L. (A: leaf phenotypic trait level, B: physiological level).

The correlations between the euclidean distance and geographical distance of the 13 phenotypic parameters and 3 physiological parameters of the 20 *R*. *pseudoacacia* provenances were analyzed. The results are shown in Fig 5 and indicate no significant correlations between the tested traits and the geographical distance of *R*. *pseudoacacia* at either leaf phenotypic or physiological levels.

### Elite tree selection based on different breeding goals

According to the above analysis, 13 leaf phenotypic traits showed abundant variation in each provenance, which is helpful for breeding improved *R*. *pseudoacacia* plants for ornamental use and food production. Because of the significant positive correlations between CLL, LA, and LN, these three characteristics could also reflect the quantity and quality of *R*. *pseudoacacia* leaves. Therefore, when the above three traits were combined for respect to food-based breeding objectives, 40 elite trees were selected after the 214 accessions were analyzed (Table 6).

**Table 6:** Selection of superior trees from *R*. *pseudoacacia* L. geographical populations based on 13 leaf phenotypic traits.

Traits	Mean	Selection of superior tree based on the CCL, LN and LA (n = 40)
Compound Leaf Length (CLL)	27.85±0.21	31.08±0.41
Compound Leaf Width (CLW)	11.25±0.10	12.86±0.15
Compound Leaf Length/Width (CLL/CLW)	2.51±0.02	2.80±0.05
Compound Petiole Length (CPL)	3.74±0.03	4.26±0.08
Leaflet Length (LL)	6.889±0.053	7.670±0.085
Leaflet Width (LW)	2.997±0.023	3.322±0.031
Leaflet Length/Width (LL/LW)	2.316±0.015	2.554±0.033
Leaflet Area (LA)	14.958±0.208	17.93±0.323
Leaflet Perimeter (LPM)	15.202±0.117	16.946±0.209
Leaflet Circularity (LC)	76.751±0.229	78.866±0.345
Leaflet Pairs (LP)	7.807±0.066	8.467±0.077
Leaflet Number (LN)	16.566±0.130	17.894±0.153
Petiole Angle (PA)	57.737±0.741	67.106±1.786

Analysis of the three physiological traits, especially Spro and PRO, provided the basis for the selection of high-quality resistance resources for *R*. *pseudoacacia*. Firstly, based on the evaluation of PRO, sixty-three excellent trees (29.439%) were selected. Secondly, based on the evaluation of Spro, eighty-four excellent trees (39.252%) were selected, whose Spro content was approximately 1.5 times that in the original provenances. Lastly, based on the evaluation of PRO and Spro, thirty excellent trees were obtained (14.019%), whose PRO and Spro contents were 1.8 times those of the original provenances, and the other two indicators also increased compared with those of the original provenances, indicating that the stress resistance of these elite trees increased at the original population level (Table 7). S3 and S4 Tables in S1 File list the specific names of these elite trees (S3 and S4 Tables in S1 File).

**Table 7:** Selection of superior trees from *R*. *pseudoacacia* L. geographical populations based on 3 physiological indexes.

Traits	Mean	Selection of superior trees based on PRO	Selection of superior trees based on Spro	Selection of superior trees based on PRO and Spro
Number	214	63	84	30
Chlorophyll Content-SPAD value (Chl)	36.05±0.23	36.49±0.47	36.21±0.37	36.38±0.67
Soluble Protein Content (Spro)	899.363±29.628	973.407±59.148	1314.885±42.891	1371.431±66.715
Proline Content (PRO)	32.382±1.569	55.067±3.980	37.019±2.695	58.740±5.612

## Discussion

Plant variation is closely related to the genetic characteristics of plants and their growth environment. In general, the larger the distribution range of a tree species, the greater the variation, and the smaller the distribution range, the smaller the variation [46, 47]. Morphological variation is an important part of genetic variation; the greater the area in which a tree species is distributed, the larger its genetic variation, leaf phenotypic and physiological differences [48, 49].

### Analysis of the variation characteristics of the leaf phenotypic and physiological characteristics of different *Robinia pseudoacacia* L. provenances

In this study, different *R*. *pseudoacacia* provenances were planted at the same site to reduce environmental variation. Our results showed that all black locust traits measured in the field varied among the provenances. Thirteen leaf phenotypic traits and 3 physiological indexes for black locust showed significant differences among 20 different provenances. Among all provenances, the TN provenance presented the largest coefficient of variation of phenotypic traits. And the KY provenance presented the largest coefficient of variation of physiological indexes of all and also the maximum of all 16 tested parameters. The LA, PA, and Spro in the KS provenance were largest, conversely, its CLL/CLW, LL/LW, LC, LP, LN, Chl, and PRO were the smallest. The degree of variation among different traits within the one provenance was diverse, indicating an imbalance in the degree of variation of leaf phenotypic traits and physiological indexes between different provenances of *R*. *pseudoacacia*. At the leaf trait level, different *R*. *pseudoacacia* provenances exhibited significant differences, consistent with the results of Granata et al. regarding *Acer campestre* leaf area morphological characteristics [50].

At the physiological level, compared to Spro and PRO, Chl had the smallest variation coefficient, with maximum-to-minimum ratios of 5.167% (Spro vs Chl) and 5.128% (PRO vs Chl). The large difference between these different physiological indicators may be due to the data obtained by the portable SPAD-502 Plus chlorophyll meter. Factors such as plant variety (genotype), environmental conditions, planting density, and nutrient conditions can affect SPAD values [51]. Using a spectrophotometric method compared to the portable chlorophyll meter SPAD-502 method, a study by Wang et al. showed a high coefficient of variation of the main greening tree species in China’s northwestern Liaoning Province [52].

It is commonly held that a coefficient of variation of traits greater than 10% represents a large difference between individuals and equates to a rich variation in traits [53]. In our research, the coefficients of variation of 13 indexes were higher than 10%, with an average total coefficient of variation of 17.924%, showing abundant variation in leaf phenotypic and physiological indexes of black locust trees, which is the basis of species selection. This variability is consistent with the findings of previous studies of the national *R*. *pseudoacacia* fine variety bases in Ji, Shanxi Ji, which showed a rich diversity in leaf phenotypic traits among 96 genotypes [38]. In addition, the degree of variation of LC and PRO was relatively large, and the degree of variation of LC was small, indicating that the same traits were affected differently in the same habitat or that different traits were affected in the same habitat. This may be related to the black locust’s the inherent genetic factors and to the influence of environmental factors [46, 54].

The differentiation coefficient of the tested indexes in our results showed that the main variation of black locust were intra-provenance variation. This is consistent with the results of previous studies on black locust [43] and other plant species [55, 56], which reveals a large potential for the selection of individual variation and can provide potential opportunities for black locust genetic improvement and germplasm preservation.

Correlation coefficients can be used to reveal the relationships between measured traits and thus greatly influence selections as part of breeding strategies [57, 58]. For all the tested parameters, compound leaf and leaflet traits generally revealed moderate and strong relationships, respectively. In particular, relationships between LA, LPM, and CLL as well as between CLW, LL, and LW have been are significantly positively correlated in most studies, such as those involving *Salix psammophila* [59] and *Phoebe bournei* [60]. Moreover, LL, LW, LL/LW, LA, LPM and LP were significantly negatively correlated with CLL/CLW, indicating leaflet traits were greatly affected by compound leaf shape. For black locust, comprehensive assessment of compound and leaflet traits were able to truly respond to the phenotypic traits of leaves. In addition, leaf size and shape can effectively reflect changes in the plant’s natural environment and adjust morphologically to water evaporation and heat loss to adapt to the environment. The CLL/CLW, LL/LW, LA, LMP, and LC traits of black locust leaves could also reflect the adaptation to the growth environment to a certain extent. For the above reasons, most leaf traits of black locust exhibited complex relationships. Among the three physiological indexes, PRO had a significant positive correlation with Spro, which may be due to the Chl value obtained by the chlorophyll meter SPAD-502 instead of values to values obtained spectrophotometrically.

Principal component analysis is a multivariate technique widely used for dimension reduction, that is, analyzing multiple related variables of one or a few comprehensive indicators [61]. In our research, leaf traits and physiological parameters were analyzed by principal component analysis. The cumulative contribution rate of the first three principal components was 65.033%, which was lower than that for the *R*. *pseudoacacia* germplasm in Shanxi and *Phoebe bournei*. Possible explanations for these results could be the weak correlations between these traits and/or the differences in the number and types of measured parameters [3, 38, 60]. Furthermore, from eigenvalue and variance contribution rate, traits such as compound length, width, petiole size, leaflet length, leaflet size, leaflet circumference and leaflet area are the main factors in the phenotypic difference of black locust samples. And Srpo and PRO are the main factors in the physiological difference of black locust samples. The above traits can be focused on in the actual breeding.

### Mantel’s test of phenotypic and physiological characteristics of different *Robinia pseudoacacia* L. provenances

Cluster analysis is a mathematical method used to find similarities between measured indexes/materials used in a group by revealing the real categories of the population and reducing the number of data points [62]. Multiple test parameters were therefore divided into dominant groups by cluster analysis, the most common and most effective classification method. The sixteen indicators could be classified into four groups, as shown in Fig 4. After comprehensive evaluations were performed, group II mainly reflected physiological indexes, and groups I and III mainly reflected leaf phenotypic characteristics of *R*. *pseudoacacia* leaves. Indicators I, II and III were the preferred test indicators to achieve the breeding objectives for practical production applications, including stress resistance, ornamental value and food production. Our results are consistent with those of many previous studies on morphological variation in *Paeonia rockii*. Four categories were divided based on 12 fruit traits, and group II was screened to maximize the economic yield per plant [63].

The results of Mantel’s test were similar to the clustering results based on euclidean distance, revealing a nonsignificant geographical variation pattern of phenotypic and physiological parameters. This is consistent with the results of previous studies of not only black locust via molecular markers such SSRs [43] and ISSRs [64] and via allozymes [65, 66] but also of other tree species, such as *Prosopis alba* [67]. Possible explanations for these results are that the collection range of provenances is broad while the numbers are small and/or black locust has migrated to its present-day range more recently.

### Elite tree selection of different *Robinia pseudoacacia* L. provenances

Phenotypic changes following a change in natural selection are particularly important for undergoing continuous adaptation [68]; this reflects the ability of plants to grow normally in nature and indicates an ability to protect the environment. Excellent tree selection is a basic method for genetic improvement of forest trees; this method involves selecting individuals with relatively good comprehensive leaf phenotypic traits after comparisons with other trees under the same site conditions [69]. Some tree breeding programs aim to select a group of black locust trees that could be used to improve ornamental quality, tolerance to soil infertility and food production for livestock. For practical applications in the present study, forty and thirty elite trees were selected according to their aggregate indicators, that were significantly correlated (18.692% and 14.019% of the total sample for three-leaf phenotypic traits (CLL, LA, and LN) and two physiological indexes (PRO and Spro), respectively. The selection rate in our study was lower than that of a comprehensive scoring method for selecting *Taxodium distichum* by Wang et al. [70]. Different elite trees were selected based on different evaluation indexes, methods and breeding objectives, resulting in different selection rates.

## Conclusions

The present study showed the following: (1) LC and LA exhibited opposite variations at the phenotypic level, of which LC had the highest stability; (2) when the differentiation coefficients of four compound leaves, nine leaflets and three physiological traits were compared the differentiation level of leaflet traits was higher than that of the other two types of indexes; (3) the variation of test traits is mainly attributed to differences within provenances, although the variation between provenances could not be ignored; and (4) there was a nonsignificant geographical variation pattern of phenotypic and physiological parameters. Suggestions and elite tree resources for germplasm preservation strategies and efficient breeding are provided to preserve the genetic resources of *R*. *pseudoacacia*.

## Supporting information

S1 File (DOCX)
