# Supplementary material for: Variation of phenotypic and physiological traits of Robinia pseudoacacia L. from 20 provenances
Source: PLoS One. 2022 Jan 5;17(1):e0262278. doi: 10.1371/journal.pone.0262278 (PMC8730420; doi:10.1371/journal.pone.0262278)
Supplement: S1 File — (DOCX) [file pone.0262278.s001.docx]

**S1 File**

**S1 Table.** Coefficient of variation (*CV %*) of phenotypic traits of different *Robinia pseudoacacia* L. geographical populations

| Population | CCL (cm) | CLW (cm) | CLL/CLW | CPL (cm) | LL (cm) | LW (cm) | LL/LW | LA (cm^2^) | LPM (cm) | LC (%) | LP（pair） | LN（piece） | PA (°) | Chl（SPAD-value） | SPro（μg·ml^-1^） | PRO（μg·g^-1^） | Mean |
| --- | --- | --- | --- | --- | --- | --- | --- | --- | --- | --- | --- | --- | --- | --- | --- | --- | --- |
| VA | 6.435 | 13.096 | 13.191 | 9.467 | 12.590 | 11.129 | 10.384 | 21.613 | 11.945 | 4.965 | 9.214 | 8.676 | 25.544 | 9.859 | 45.325 | 63.731 | 17.323 |
| WV | 10.613 | 13.872 | 8.749 | 10.485 | 11.226 | 9.931 | 9.675 | 20.547 | 11.950 | 4.979 | 11.436 | 10.370 | 18.187 | 9.575 | 47.677 | 85.305 | 18.411 |
| NC | 9.221 | 9.528 | 14.114 | 8.990 | 6.620 | 4.604 | 6.205 | 8.529 | 6.674 | 3.389 | 14.469 | 13.607 | 10.081 | 15.013 | 10.076 | 22.456 | 10.224 |
| GA | 9.849 | 12.124 | 9.532 | 8.757 | 13.039 | 14.065 | 6.458 | 25.974 | 15.020 | 3.610 | 11.223 | 10.365 | 12.644 | 6.624 | 58.118 | 26.994 | 15.275 |
| MD | 8.946 | 16.732 | 14.982 | 19.386 | 12.959 | 11.029 | 4.212 | 22.074 | 12.667 | 1.564 | 9.661 | 9.008 | 19.322 | 8.291 | 31.927 | 18.873 | 13.852 |
| PA | 15.503 | 16.584 | 9.728 | 15.374 | 14.049 | 12.441 | 7.923 | 25.049 | 12.739 | 3.360 | 11.205 | 10.581 | 18.218 | 6.105 | 50.192 | 29.735 | 16.174 |
| OH | 11.324 | 12.898 | 9.107 | 14.755 | 7.307 | 8.554 | 7.379 | 14.372 | 7.489 | 3.438 | 8.997 | 8.471 | 20.318 | 7.931 | 48.527 | 84.689 | 17.222 |
| IN | 10.935 | 10.694 | 10.771 | 12.238 | 8.601 | 11.713 | 9.511 | 18.822 | 9.870 | 4.380 | 7.967 | 7.590 | 22.501 | 7.428 | 47.628 | 44.419 | 15.317 |
| IL | 10.474 | 12.759 | 12.550 | 10.005 | 14.742 | 8.168 | 12.823 | 20.655 | 16.471 | 4.949 | 10.967 | 10.337 | 13.005 | 7.977 | 44.897 | 34.285 | 15.317 |
| KY | 10.886 | 12.093 | 13.687 | 10.740 | 8.161 | 11.199 | 8.181 | 18.084 | 8.358 | 3.833 | 12.106 | 11.133 | 9.398 | 10.089 | 45.115 | 103.155 | 18.514 |
| TN | 15.614 | 15.336 | 10.122 | 15.553 | 16.596 | 14.414 | 12.660 | 29.745 | 14.087 | 6.028 | 8.954 | 8.439 | 24.695 | 8.253 | 42.800 | 30.387 | 17.105 |
| MS | 13.102 | 15.834 | 22.833 | 9.222 | 9.321 | 12.879 | 17.263 | 16.037 | 5.347 | 7.369 | 21.350 | 20.027 | 21.631 | 12.736 | 14.413 | 14.752 | 14.632 |
| AL | 8.759 | 6.339 | 7.629 | 14.749 | 8.022 | 7.947 | 4.458 | 13.777 | 8.637 | 2.401 | 13.675 | 12.778 | 9.464 | 7.211 | 46.559 | 40.364 | 13.298 |
| MS/AL | 4.425 | 5.936 | 4.970 | 8.913 | 5.302 | 7.529 | 7.781 | 9.820 | 5.729 | 3.044 | 9.935 | 9.125 | 12.206 | 7.726 | 30.833 | 35.867 | 10.571 |
| IA | 9.206 | 11.297 | 7.616 | 3.977 | 14.457 | 10.581 | 8.527 | 24.412 | 14.556 | 4.180 | 12.047 | 10.830 | 17.352 | 8.356 | 31.166 | 45.128 | 14.606 |
| MO | 9.541 | 10.623 | 10.571 | 10.855 | 12.031 | 8.711 | 8.094 | 19.317 | 11.775 | 4.118 | 11.938 | 11.222 | 14.731 | 7.000 | 37.188 | 37.847 | 14.098 |
| KS | 13.289 | 16.513 | 2.881 | 5.039 | 15.599 | 14.333 | 1.317 | 29.946 | 15.246 | 0.229 | 1.188 | 1.131 | 26.621 | 2.359 | 88.020 | 5.379 | 14.943 |
| OK | 11.442 | 8.257 | 10.633 | 12.228 | 9.505 | 12.085 | 10.643 | 19.515 | 9.842 | 3.948 | 6.992 | 6.471 | 9.015 | 10.929 | 26.428 | 44.924 | 13.304 |
| AR | 13.794 | 14.557 | 9.780 | 9.213 | 8.631 | 8.262 | 6.249 | 15.779 | 5.585 | 0.554 | 7.669 | 7.000 | 19.005 | 3.351 | 52.110 | 28.500 | 13.127 |
| CN | 10.795 | 12.115 | 10.499 | 13.629 | 10.078 | 10.868 | 9.496 | 17.916 | 10.831 | 4.485 | 10.158 | 9.471 | 22.394 | 8.381 | 48.258 | 50.324 | 16.231 |
| Mean | 10.708 | 12.359 | 10.697 | 11.179 | 10.942 | 10.522 | 8.462 | 19.599 | 10.741 | 3.741 | 10.558 | 9.832 | 17.317 | 8.260 | 42.363 | 42.356 | 14.977 |

**S2 Table.** Correlations between sixteen traits in different *Robinia pseudoacacia* L. geographical provenances

|  | CLL | CLW | CLL/CLW | CPL | LL | LW | LL/LW | LA | LPM | LC | LP | LN | PA | Chl | SPro | PRO |
| --- | --- | --- | --- | --- | --- | --- | --- | --- | --- | --- | --- | --- | --- | --- | --- | --- |
| CLL | 1.000 |  |  |  |  |  |  |  |  |  |  |  |  |  |  |  |
| CLW | 0.554** | 1.000 |  |  |  |  |  |  |  |  |  |  |  |  |  |  |
| CLL/CLW | 0.341** | -0.580** | 1.000 |  |  |  |  |  |  |  |  |  |  |  |  |  |
| CPL | 0.500** | 0.402** | 0.019 | 1.000 |  |  |  |  |  |  |  |  |  |  |  |  |
| LL | 0.578** | 0.804** | -0.317** | 0.415** | 1.000 |  |  |  |  |  |  |  |  |  |  |  |
| LW | 0.488** | 0.580** | -0.178** | 0.507** | 0.646** | 1.000 |  |  |  |  |  |  |  |  |  |  |
| LL/LW | 0.112 | 0.260** | -0.155* | -0.106 | 0.415** | -0.420** | 1.000 |  |  |  |  |  |  |  |  |  |
| LA | 0.593** | 0.758** | -0.262** | 0.499** | 0.898** | 0.905** | -0.012 | 1.000 |  |  |  |  |  |  |  |  |
| LPM | 0.573** | 0.774** | -0.293** | 0.435** | 0.955** | 0.746** | 0.245** | 0.928** | 1.000 |  |  |  |  |  |  |  |
| LC | -0.045 | -0.179** | 0.125 | 0.072 | -0.341** | 0.404** | -0.891** | 0.081 | -0.192** | 1.000 |  |  |  |  |  |  |
| LP | 0.456** | -0.023 | -0.482** | -0.081 | 0.031 | -0.132 | 0.204** | -0.040 | -0.016 | -0.099 | 1.000 |  |  |  |  |  |
| LN | 0.466** | -0.013 | 0.481** | -0.076 | 0.036 | -0.127 | 0.205** | -0.034 | -0.011 | -0.101 | 0.999** | 1.000 |  |  |  |  |
| PA | -0.034 | -0.155* | 0.103 | -0.165* | -0.200** | 0.018 | -0.259** | -0.097 | -0.152* | 0.235** | -0.023 | -0.026 | 1.000 |  |  |  |
| Chl | 0.056 | 0.015 | 0.048 | -0.005 | 0.043 | -0.003 | 0.048 | 0.022 | 0.025 | 0.002 | 0.178** | 0.172* | -0.163* | 1.000 |  |  |
| SPro | -0.123 | 0.001 | -0.114 | -0.174* | -0.057 | -0.149* | 0.105 | -0.103 | -0.092 | -0.067 | 0.117 | 0.113 | 0.069 | -0.036 | 1.000 |  |
| PRO | -0.174* | -0.117 | -0.043 | -0.178** | -0.070 | -0.194** | 0.145* | -0.143* | -0.106 | -0.121 | 0.030 | 0.023 | 0.106 | 0.058 | 0.255** | 1.000 |

Note: CLL: Compound leaf length; CLW: Compound leaf width; CLF/CLW: Compound leaf length/width; CPL: Compound petiole length; LL: Leaflet length; LW: Leaflet width；LL/LW: Leaflet length/width；LA：Leaflet area；LPM：Leaflet perimeter; LC：Leaflet circularity; LP：Leaflet pairs; LN：Leaflet numbers; PA：Petiole angle; Chl: Chlorophyll content-SPAD value; Spro: Soluble protein content; PRO: Protein content. “*” Correlation is significant at the 0.05 level (two side); “**” Correlation is highly significant at the 0.01 level (two side).

**S3 Table.** Names of *Robinia pseudoacacia* L. trees based on phenotypic trait selection

| Traits | Name of solid seedlings | | | | | | | | | |
| --- | --- | --- | --- | --- | --- | --- | --- | --- | --- | --- |
|  | 2 | 3 | 172 | 173 | 242 | 77 | 81 | 83 | 175 | 195 |
| phenotype | 65 | 22 | 67 | 68 | 72 | 30 | 210 | 36 | 38 | 39 |
|  | 100 | 40 | 41 | 44 | 140 | 215 | 219 | 231 | 150 | 152 |
|  | 155 | 157 | 46 | 53 | 135 | 162 | 168 | YA28 | YA39 | Q41 |

**S4 Table.** Names of *Robinia pseudoacacia* L. trees based on physiological trait selection

| Tratis | Name of solid seedlings | | | | | | | | | |
| --- | --- | --- | --- | --- | --- | --- | --- | --- | --- | --- |
| PRO | 3 | 172 | 174 | 240 | 242 | 9 | 13 | 15 | 74 | 80 |
|  | 82 | 83 | 84 | 85 | 86 | 87 | 192 | 61 | 16 | 29 |
|  | 32 | 204 | 207 | 34 | 35 | 38 | 95 | 96 | 98 | 101 |
|  | 102 | 103 | 104 | 40 | 44 | 117 | 118 | 119 | 88 | 89 |
|  | 139 | 144 | 213 | 219 | 233 | 234 | 235 | 236 | 147 | 148 |
|  | 149 | 157 | 46 | 48 | 49 | 53 | 56 | 57 | 109 | 113 |
|  | 137 | CK | YC21 |  |  |  |  |  |  |  |
| SPro | 1 | 170 | 171 | 174 | 238 | 240 | 241 | 242 | 244 | 9 |
|  | 11 | 74 | 76 | 78 | 80 | 82 | 83 | 86 | 87 | 191 |
|  | 192 | 194 | 16 | 72 | 73 | 26 | 204 | 206 | 208 | 210 |
|  | 95 | 96 | 97 | 98 | 99 | 100 | 101 | 102 | 108 | 44 |
|  | 117 | 122 | 88 | 89 | 94 | 143 | 212 | 213 | 218 | 219 |
|  | 220 | 221 | 197 | 202 | 231 | 232 | 236 | 237 | 224 | 225 |
|  | 226 | 147 | 149 | 155 | 56 | 58 | 112 | 114 | 131 | 132 |
|  | 136 | 138 | CK | YA12 | YA28 | YA39 | YC9 | YC21 | Y23 | YC28 |
|  | P | Q21 | Q28 | Q41 |  |  |  |  |  |  |
| PRO, SPro | 174 | 240 | 242 | 9 | 74 | 80 | 82 | 83 | 86 | 87 |
|  | 192 | 16 | 204 | 95 | 96 | 98 | 101 | 102 | 44 | 117 |
|  | 88 | 89 | 213 | 219 | 236 | 147 | 149 | 56 | CK | YC21 |

**S5 Table. Phenotypic traits data of different provenances.**

| No. | Provenances | CLL | CLW | CLL/CLW | CPL | LL | LW | LL/LW | LA | LPM | LC | LP | LN | PA |
| --- | --- | --- | --- | --- | --- | --- | --- | --- | --- | --- | --- | --- | --- | --- |
| 1 | VA | 23.89 | 7.91 | 3.04 | 3.79 | 4.607 | 2.548 | 1.810 | 8.599 | 10.578 | 84.829 | 6.833 | 14.583 | 61.91 |
| 2 | VA | 31.50 | 11.29 | 2.84 | 3.92 | 7.268 | 3.286 | 2.219 | 17.475 | 15.942 | 79.689 | 7.833 | 16.667 | 61.29 |
| 3 | VA | 30.39 | 12.43 | 2.46 | 3.78 | 7.659 | 2.894 | 2.652 | 15.692 | 16.332 | 71.059 | 7.833 | 16.583 | 61.41 |
| 4 | VA | 27.38 | 13.03 | 2.11 | 3.13 | 7.582 | 2.789 | 2.721 | 15.353 | 16.095 | 72.633 | 7.667 | 16.333 | 39.89 |
| 5 | VA | 27.60 | 11.87 | 2.35 | 4.00 | 6.737 | 3.002 | 2.246 | 14.338 | 14.614 | 76.839 | 7.000 | 15.000 | 42.58 |
| 6 | VA | 28.79 | 12.83 | 2.25 | 3.53 | 7.081 | 2.991 | 2.374 | 15.336 | 15.320 | 76.527 | 7.667 | 16.333 | 44.82 |
| 7 | VA | 30.33 | 12.29 | 2.49 | 3.70 | 8.452 | 3.541 | 2.389 | 21.651 | 18.264 | 76.347 | 7.083 | 15.167 | 61.53 |
| 8 | VA | 28.75 | 10.29 | 2.80 | 3.75 | 6.353 | 2.593 | 2.466 | 12.025 | 14.089 | 76.067 | 8.083 | 17.167 | 52.91 |
| 9 | VA | 29.13 | 9.73 | 3.02 | 3.62 | 6.278 | 2.715 | 2.325 | 12.154 | 13.728 | 76.319 | 8.583 | 18.167 | 50.12 |
| 10 | VA | 29.83 | 12.03 | 2.48 | 3.71 | 7.004 | 3.270 | 2.153 | 16.663 | 15.580 | 80.208 | 8.583 | 18.167 | 83.17 |
| 11 | VA | 28.38 | 11.61 | 2.45 | 3.45 | 7.429 | 3.299 | 2.268 | 17.018 | 15.897 | 75.041 | 7.833 | 16.667 | 75.73 |
| 12 | VA | 29.10 | 13.00 | 2.26 | 3.88 | 7.883 | 3.379 | 2.338 | 19.026 | 17.157 | 75.860 | 7.500 | 16.000 | 58.18 |
| 13 | VA | 29.66 | 13.05 | 2.29 | 3.93 | 7.556 | 3.276 | 2.321 | 17.834 | 16.635 | 77.035 | 6.750 | 14.500 | 35.28 |
| 14 | VA | 27.23 | 13.32 | 2.05 | 4.00 | 7.453 | 2.638 | 2.842 | 13.627 | 17.341 | 67.922 | 7.333 | 15.667 | 43.52 |
| 15 | VA | 26.97 | 15.23 | 1.78 | 4.06 | 9.000 | 3.520 | 2.573 | 23.120 | 19.210 | 74.656 | 7.167 | 15.250 | 43.05 |
| 16 | VA | 29.18 | 11.32 | 2.60 | 3.89 | 6.778 | 3.001 | 2.257 | 14.296 | 14.672 | 75.961 | 8.833 | 18.667 | 46.63 |
| 17 | VA | 29.22 | 12.71 | 2.30 | 4.07 | 7.139 | 3.151 | 2.270 | 16.546 | 15.801 | 78.584 | 8.250 | 17.417 | 64.61 |
| 18 | VA | 27.16 | 11.99 | 2.29 | 4.93 | 7.221 | 3.633 | 2.005 | 19.271 | 17.316 | 81.795 | 6.083 | 13.167 | 55.54 |
| 19 | VA | 25.29 | 10.86 | 2.34 | 3.45 | 6.901 | 2.706 | 2.559 | 13.356 | 14.782 | 73.607 | 7.917 | 16.833 | 86.65 |
| 20 | WV | 23.92 | 10.29 | 2.33 | 3.47 | 6.151 | 3.020 | 2.052 | 13.625 | 13.864 | 81.898 | 6.583 | 14.167 | 57.42 |
| 21 | WV | 22.24 | 8.16 | 2.73 | 3.43 | 5.437 | 2.500 | 2.189 | 9.211 | 11.720 | 74.203 | 7.667 | 16.333 | 34.11 |
| 22 | WV | 28.73 | 11.70 | 2.47 | 3.64 | 6.743 | 2.965 | 2.274 | 14.459 | 14.580 | 77.915 | 8.417 | 17.750 | 48.98 |
| 23 | WV | 26.88 | 11.75 | 2.30 | 3.84 | 7.133 | 2.836 | 2.521 | 14.623 | 15.498 | 74.891 | 6.917 | 14.833 | 50.29 |
| 24 | WV | 23.54 | 8.97 | 2.64 | 3.40 | 6.523 | 2.628 | 2.543 | 11.442 | 13.859 | 69.222 | 7.083 | 15.000 | 43.20 |
| 25 | WV | 24.58 | 9.94 | 2.48 | 3.70 | 6.354 | 2.458 | 2.591 | 10.791 | 13.321 | 70.493 | 8.000 | 16.750 | 65.07 |
| 26 | WV | 24.66 | 10.18 | 2.40 | 4.03 | 6.435 | 2.885 | 2.234 | 13.582 | 14.033 | 79.446 | 8.667 | 18.083 | 52.73 |
| 27 | WV | 26.04 | 11.08 | 2.39 | 3.06 | 7.485 | 2.893 | 2.600 | 15.639 | 16.501 | 73.493 | 7.917 | 16.750 | 46.30 |
| 28 | WV | 30.51 | 12.32 | 2.49 | 3.83 | 7.048 | 2.905 | 2.434 | 14.921 | 15.391 | 76.391 | 8.917 | 18.583 | 49.71 |
| 29 | WV | 29.97 | 12.55 | 2.39 | 3.43 | 7.166 | 3.507 | 2.049 | 18.323 | 17.439 | 81.647 | 8.500 | 17.917 | 53.99 |
| 30 | WV | 30.53 | 11.79 | 2.60 | 4.38 | 6.823 | 3.095 | 2.207 | 15.437 | 15.224 | 79.449 | 7.083 | 15.167 | 42.98 |
| 31 | WV | 28.43 | 9.37 | 3.08 | 3.65 | 7.019 | 2.886 | 2.437 | 14.560 | 16.232 | 75.155 | 8.667 | 18.250 | 54.63 |
| 32 | WV | 26.38 | 11.56 | 2.29 | 3.67 | 6.493 | 2.659 | 2.451 | 12.393 | 14.003 | 74.634 | 8.000 | 17.000 | 38.26 |
| 33 | WV | 29.75 | 12.73 | 2.36 | 3.43 | 7.488 | 2.743 | 2.742 | 15.205 | 16.384 | 73.529 | 7.917 | 16.833 | 37.73 |
| 34 | WV | 31.38 | 12.40 | 2.55 | 3.47 | 8.058 | 2.779 | 2.903 | 16.180 | 17.350 | 69.908 | 7.583 | 16.083 | 54.86 |
| 35 | WV | 29.55 | 14.43 | 2.05 | 3.38 | 8.725 | 3.528 | 2.478 | 22.735 | 19.088 | 77.044 | 9.333 | 19.583 | 54.68 |
| 36 | WV | 24.75 | 9.71 | 2.56 | 3.20 | 5.882 | 2.870 | 2.048 | 12.371 | 13.007 | 81.363 | 6.583 | 14.167 | 66.66 |
| 37 | WV | 28.96 | 12.55 | 2.33 | 4.08 | 7.376 | 3.237 | 2.285 | 17.087 | 15.809 | 76.921 | 6.667 | 14.333 | 41.87 |
| 38 | WV | 22.65 | 10.15 | 2.24 | 2.90 | 6.017 | 2.583 | 2.340 | 11.468 | 13.048 | 78.441 | 8.250 | 17.333 | 64.36 |
| 39 | WV | 27.27 | 10.37 | 2.64 | 2.98 | 6.500 | 2.850 | 2.289 | 13.701 | 14.405 | 79.438 | 9.667 | 19.917 | 57.64 |
| 40 | NC | 33.44 | 10.71 | 3.18 | 3.84 | 7.297 | 3.375 | 2.161 | 17.447 | 16.251 | 76.984 | 9.250 | 19.500 | 54.29 |
| 41 | NC | 26.42 | 11.71 | 2.27 | 3.47 | 6.698 | 3.326 | 2.020 | 16.346 | 15.120 | 81.843 | 6.917 | 14.833 | 62.41 |
| 42 | NC | 28.43 | 12.26 | 2.33 | 4.10 | 7.274 | 3.364 | 2.170 | 17.776 | 15.944 | 79.653 | 7.083 | 15.083 | 65.92 |
| 43 | NC | 28.57 | 12.63 | 2.27 | 4.28 | 7.902 | 3.261 | 2.429 | 18.186 | 17.865 | 73.939 | 6.583 | 14.167 | 52.35 |
| 44 | NC | 26.23 | 9.69 | 2.75 | 4.52 | 7.028 | 3.095 | 2.283 | 15.805 | 15.403 | 78.102 | 6.583 | 14.167 | 53.41 |
| 45 | NC | 27.75 | 11.07 | 2.54 | 4.13 | 6.607 | 3.019 | 2.186 | 14.407 | 14.998 | 78.477 | 8.250 | 17.500 | 53.24 |
| 46 | GA | 29.08 | 13.17 | 2.23 | 3.72 | 8.355 | 3.797 | 2.207 | 22.674 | 19.310 | 77.391 | 7.667 | 16.333 | 49.23 |
| 47 | GA | 24.43 | 10.97 | 2.23 | 3.85 | 6.300 | 2.613 | 2.443 | 11.777 | 13.654 | 74.549 | 7.333 | 15.500 | 52.78 |
| 48 | GA | 25.75 | 11.96 | 2.17 | 3.63 | 7.084 | 3.263 | 2.182 | 17.143 | 16.555 | 81.274 | 6.083 | 13.167 | 62.97 |
| 49 | GA | 29.33 | 11.11 | 2.68 | 3.63 | 7.883 | 3.318 | 2.381 | 18.254 | 18.073 | 73.824 | 6.583 | 14.167 | 57.52 |
| 50 | GA | 27.71 | 10.83 | 2.56 | 3.28 | 6.038 | 2.612 | 2.321 | 11.286 | 13.349 | 76.470 | 8.417 | 17.833 | 67.28 |
| 51 | GA | 33.04 | 14.63 | 2.27 | 4.28 | 7.631 | 3.532 | 2.166 | 19.346 | 17.076 | 78.135 | 8.000 | 16.750 | 68.96 |
| 52 | GA | 29.27 | 10.96 | 2.69 | 4.08 | 6.170 | 3.047 | 2.020 | 13.573 | 13.710 | 80.495 | 6.917 | 14.750 | 66.43 |
| 53 | MD | 26.71 | 9.03 | 2.96 | 2.88 | 5.262 | 2.463 | 2.147 | 9.431 | 11.795 | 79.032 | 8.083 | 17.000 | 51.74 |
| 54 | MD | 27.32 | 10.57 | 2.61 | 3.35 | 6.767 | 2.879 | 2.360 | 14.051 | 14.633 | 76.478 | 7.583 | 16.000 | 65.15 |
| 55 | MD | 24.67 | 11.82 | 2.10 | 3.88 | 6.911 | 3.087 | 2.251 | 15.426 | 15.798 | 78.320 | 6.417 | 13.833 | 49.09 |
| 56 | MD | 31.45 | 13.27 | 2.37 | 4.35 | 7.632 | 3.323 | 2.295 | 18.179 | 16.793 | 76.850 | 8.000 | 17.000 | 40.00 |
| 57 | MD | 27.58 | 9.17 | 3.03 | 4.73 | 6.708 | 3.141 | 2.144 | 15.093 | 15.018 | 79.073 | 8.250 | 17.500 | 43.74 |
| 58 | PA | 28.80 | 11.91 | 2.44 | 3.85 | 7.038 | 3.140 | 2.247 | 16.059 | 15.532 | 78.786 | 7.500 | 15.833 | 71.96 |
| 59 | PA | 27.70 | 9.93 | 2.88 | 3.45 | 6.324 | 2.929 | 2.150 | 13.378 | 13.896 | 78.211 | 7.750 | 16.500 | 61.96 |
| 60 | PA | 23.94 | 8.97 | 2.70 | 3.37 | 6.009 | 2.631 | 2.286 | 11.356 | 13.256 | 77.065 | 6.750 | 14.667 | 50.82 |
| 61 | PA | 30.71 | 11.18 | 2.76 | 4.54 | 7.208 | 3.054 | 2.378 | 15.936 | 15.635 | 76.893 | 7.417 | 15.750 | 57.32 |
| 62 | PA | 36.67 | 12.73 | 2.90 | 3.65 | 8.267 | 3.126 | 2.672 | 19.012 | 17.731 | 74.296 | 9.083 | 19.167 | 53.23 |
| 63 | PA | 22.50 | 8.79 | 2.62 | 3.68 | 6.131 | 2.394 | 2.571 | 10.184 | 13.739 | 71.108 | 7.083 | 15.083 | 51.73 |
| 64 | PA | 25.08 | 8.79 | 2.88 | 3.31 | 6.009 | 2.824 | 2.137 | 12.128 | 13.426 | 78.887 | 8.417 | 17.500 | 53.43 |
| 65 | PA | 28.46 | 12.04 | 2.37 | 3.93 | 6.915 | 3.245 | 2.143 | 16.417 | 15.333 | 80.705 | 8.917 | 18.833 | 55.62 |
| 66 | PA | 35.25 | 12.78 | 2.76 | 5.30 | 7.471 | 3.584 | 2.091 | 19.159 | 16.631 | 79.397 | 8.833 | 18.667 | 80.98 |
| 67 | PA | 29.16 | 12.64 | 2.32 | 4.21 | 8.353 | 3.534 | 2.367 | 20.912 | 17.871 | 75.297 | 7.167 | 15.333 | 54.67 |
| 68 | PA | 34.93 | 13.58 | 2.58 | 4.89 | 8.066 | 3.508 | 2.305 | 20.026 | 17.869 | 75.865 | 7.500 | 16.000 | 50.28 |
| 69 | PA | 27.38 | 9.38 | 2.93 | 3.94 | 5.534 | 2.564 | 2.161 | 9.983 | 12.516 | 76.986 | 7.500 | 16.000 | 37.14 |
| 70 | PA | 32.79 | 14.43 | 2.28 | 3.68 | 8.574 | 3.353 | 2.565 | 20.574 | 18.246 | 73.417 | 8.917 | 18.833 | 54.36 |
| 71 | PA | 23.88 | 11.00 | 2.19 | 3.33 | 6.684 | 2.757 | 2.419 | 13.375 | 14.355 | 75.811 | 6.417 | 13.667 | 54.86 |
| 72 | OH | 25.08 | 10.53 | 2.40 | 4.17 | 7.278 | 3.064 | 2.392 | 15.901 | 15.905 | 75.356 | 6.583 | 14.167 | 48.13 |
| 73 | OH | 28.28 | 11.77 | 2.42 | 3.58 | 6.919 | 2.963 | 2.337 | 14.932 | 14.993 | 77.480 | 7.750 | 16.417 | 39.86 |
| 74 | OH | 24.63 | 8.80 | 2.84 | 2.98 | 5.948 | 2.651 | 2.251 | 11.223 | 13.059 | 77.023 | 8.000 | 17.000 | 56.59 |
| 75 | OH | 33.12 | 11.48 | 2.91 | 4.24 | 7.013 | 2.693 | 2.605 | 13.393 | 14.929 | 72.256 | 8.333 | 17.667 | 43.29 |
| 76 | OH | 32.91 | 14.16 | 2.34 | 4.15 | 7.661 | 3.433 | 2.254 | 18.761 | 16.846 | 77.286 | 8.333 | 17.833 | 44.36 |
| 77 | OH | 26.22 | 9.15 | 2.88 | 4.22 | 6.965 | 3.043 | 2.291 | 15.051 | 15.110 | 76.440 | 7.917 | 16.833 | 46.47 |
| 78 | OH | 25.88 | 10.86 | 2.42 | 2.81 | 6.953 | 2.605 | 2.685 | 12.954 | 14.708 | 71.251 | 7.333 | 15.667 | 68.80 |
| 79 | OH | 25.04 | 10.65 | 2.43 | 3.13 | 6.500 | 3.153 | 2.066 | 14.775 | 14.483 | 80.424 | 6.750 | 14.500 | 68.05 |
| 80 | OH | 26.08 | 11.49 | 2.28 | 3.63 | 7.167 | 3.063 | 2.346 | 15.922 | 16.186 | 77.136 | 7.167 | 15.333 | 58.68 |
| 81 | OH | 25.68 | 10.46 | 2.50 | 3.17 | 6.094 | 2.735 | 2.237 | 11.989 | 13.331 | 78.027 | 7.667 | 16.250 | 71.02 |
| 82 | OH | 29.96 | 11.02 | 2.73 | 3.58 | 6.844 | 3.030 | 2.279 | 15.080 | 14.905 | 77.996 | 8.833 | 18.583 | 55.59 |
| 83 | IN | 29.24 | 11.81 | 2.49 | 4.12 | 6.821 | 2.980 | 2.291 | 14.948 | 15.131 | 78.991 | 8.583 | 18.167 | 56.68 |
| 84 | IN | 28.85 | 10.86 | 2.67 | 4.04 | 6.608 | 3.149 | 2.102 | 14.711 | 14.507 | 78.661 | 8.917 | 18.833 | 77.08 |
| 85 | IN | 29.28 | 12.27 | 2.40 | 3.93 | 7.616 | 2.938 | 2.595 | 16.282 | 16.475 | 74.305 | 8.917 | 18.750 | 48.91 |
| 86 | IN | 27.35 | 10.86 | 2.53 | 3.89 | 7.215 | 3.116 | 2.331 | 16.497 | 16.234 | 78.007 | 8.167 | 17.333 | 54.25 |
| 87 | IN | 35.75 | 12.50 | 2.86 | 4.12 | 7.666 | 3.685 | 2.096 | 20.470 | 18.838 | 80.335 | 8.083 | 17.083 | 63.59 |
| 88 | IN | 33.83 | 13.42 | 2.53 | 4.48 | 7.434 | 3.561 | 2.089 | 19.395 | 16.495 | 81.344 | 8.000 | 17.000 | 46.76 |
| 89 | IN | 23.66 | 10.68 | 2.23 | 2.98 | 6.658 | 2.542 | 2.624 | 12.647 | 14.473 | 76.032 | 7.083 | 15.083 | 65.36 |
| 90 | IN | 27.28 | 9.77 | 2.82 | 4.48 | 6.879 | 3.137 | 2.201 | 15.382 | 15.257 | 77.261 | 8.083 | 16.833 | 54.82 |
| 91 | IN | 24.01 | 10.08 | 2.40 | 3.21 | 5.644 | 2.693 | 2.102 | 10.850 | 12.508 | 78.959 | 8.667 | 18.333 | 63.74 |
| 92 | IN | 25.13 | 11.50 | 2.19 | 3.06 | 6.585 | 2.316 | 2.863 | 10.944 | 13.810 | 70.225 | 8.583 | 18.167 | 40.58 |
| 93 | IN | 26.94 | 12.79 | 2.11 | 3.62 | 7.526 | 3.093 | 2.439 | 16.634 | 16.006 | 74.281 | 6.750 | 14.500 | 51.47 |
| 94 | IN | 28.71 | 13.02 | 2.22 | 3.58 | 7.721 | 3.313 | 2.342 | 18.357 | 16.828 | 76.165 | 8.333 | 17.667 | 82.70 |
| 95 | IN | 27.33 | 12.81 | 2.14 | 3.81 | 7.436 | 3.170 | 2.375 | 17.288 | 16.211 | 77.679 | 8.500 | 17.917 | 40.05 |
| 96 | IN | 27.59 | 10.72 | 2.60 | 3.53 | 6.461 | 2.972 | 2.175 | 13.362 | 15.243 | 76.333 | 7.583 | 16.167 | 82.51 |
| 97 | IN | 30.45 | 13.00 | 2.36 | 3.69 | 7.558 | 2.933 | 2.574 | 14.949 | 16.108 | 68.877 | 8.417 | 17.667 | 48.45 |
| 98 | IN | 29.05 | 9.64 | 3.05 | 3.16 | 6.531 | 2.610 | 2.514 | 12.139 | 13.745 | 73.122 | 9.417 | 19.833 | 72.07 |
| 99 | IN | 26.27 | 10.73 | 2.46 | 3.53 | 6.240 | 2.746 | 2.279 | 12.288 | 13.653 | 77.466 | 8.333 | 17.417 | 62.89 |
| 100 | IL | 32.58 | 12.78 | 2.58 | 4.18 | 8.861 | 3.198 | 2.786 | 20.719 | 19.540 | 72.141 | 8.750 | 18.500 | 64.22 |
| 101 | IL | 33.76 | 13.18 | 2.58 | 4.13 | 7.573 | 2.984 | 2.547 | 16.759 | 16.711 | 76.083 | 9.500 | 20.000 | 46.26 |
| 102 | IL | 25.79 | 9.47 | 2.54 | 3.48 | 5.701 | 3.113 | 1.842 | 12.487 | 12.704 | 81.258 | 8.417 | 17.833 | 61.53 |
| 103 | IL | 24.25 | 9.53 | 2.56 | 3.59 | 6.083 | 2.993 | 2.038 | 13.027 | 13.372 | 80.105 | 8.583 | 18.083 | 58.43 |
| 104 | IL | 29.33 | 12.07 | 2.44 | 4.62 | 8.526 | 3.216 | 2.662 | 18.896 | 20.412 | 69.690 | 7.917 | 16.833 | 47.55 |
| 105 | IL | 29.29 | 10.17 | 2.91 | 3.36 | 6.193 | 2.952 | 2.118 | 13.283 | 14.094 | 79.845 | 9.083 | 18.833 | 50.36 |
| 106 | IL | 33.03 | 11.91 | 2.78 | 4.06 | 6.548 | 2.796 | 2.349 | 13.000 | 14.109 | 75.687 | 8.750 | 18.500 | 62.46 |
| 107 | IL | 26.44 | 9.84 | 2.72 | 3.76 | 6.489 | 2.420 | 2.696 | 11.230 | 13.834 | 71.460 | 9.250 | 19.333 | 64.28 |
| 108 | IL | 28.57 | 10.41 | 2.76 | 3.43 | 6.467 | 2.613 | 2.480 | 12.135 | 13.953 | 74.759 | 9.167 | 19.250 | 58.84 |
| 109 | IL | 30.13 | 10.59 | 2.87 | 3.99 | 6.504 | 3.079 | 2.112 | 14.384 | 14.242 | 79.051 | 7.917 | 16.833 | 55.25 |
| 110 | IL | 33.33 | 9.00 | 3.76 | 3.56 | 5.949 | 2.765 | 2.153 | 11.730 | 13.255 | 78.431 | 11.250 | 23.333 | 53.28 |
| 111 | IL | 30.03 | 11.69 | 2.59 | 3.61 | 7.295 | 2.869 | 2.558 | 15.058 | 15.546 | 73.920 | 7.500 | 15.750 | 71.25 |
| 112 | KY | 25.39 | 9.58 | 2.70 | 3.55 | 6.803 | 2.771 | 2.448 | 13.434 | 14.526 | 73.643 | 7.583 | 16.000 | 63.22 |
| 113 | KY | 23.63 | 10.09 | 2.35 | 4.03 | 5.714 | 2.418 | 2.367 | 9.750 | 12.397 | 74.582 | 6.583 | 14.083 | 71.05 |
| 114 | KY | 33.00 | 12.46 | 2.68 | 4.48 | 6.963 | 3.030 | 2.309 | 15.164 | 15.381 | 76.950 | 7.500 | 16.000 | 75.77 |
| 115 | KY | 32.18 | 11.29 | 2.88 | 3.10 | 6.677 | 2.654 | 2.518 | 12.886 | 14.378 | 74.691 | 9.083 | 19.083 | 69.39 |
| 116 | KY | 29.78 | 10.17 | 2.96 | 4.00 | 6.738 | 2.673 | 2.535 | 13.153 | 14.447 | 75.280 | 7.917 | 16.667 | 63.28 |
| 117 | KY | 27.23 | 9.78 | 2.80 | 3.22 | 6.221 | 2.836 | 2.200 | 12.181 | 13.491 | 75.438 | 8.000 | 17.000 | 53.76 |
| 118 | KY | 23.79 | 10.73 | 2.24 | 3.57 | 6.586 | 2.656 | 2.487 | 12.567 | 14.171 | 74.647 | 6.167 | 13.333 | 66.92 |
| 119 | KY | 28.93 | 9.47 | 3.08 | 3.28 | 7.116 | 3.105 | 2.307 | 16.325 | 15.679 | 78.883 | 8.750 | 18.417 | 58.38 |
| 120 | KY | 27.01 | 11.42 | 2.38 | 3.66 | 6.974 | 2.986 | 2.347 | 14.481 | 15.978 | 74.003 | 6.750 | 14.333 | 61.05 |
| 121 | KY | 30.43 | 13.32 | 2.30 | 4.29 | 7.883 | 3.485 | 2.266 | 19.152 | 17.474 | 75.372 | 6.417 | 13.833 | 56.83 |
| 122 | KY | 29.71 | 11.98 | 2.49 | 4.13 | 6.912 | 3.250 | 2.131 | 16.483 | 15.577 | 80.860 | 7.500 | 16.167 | 67.41 |
| 123 | KY | 25.29 | 11.10 | 2.28 | 3.72 | 6.231 | 2.942 | 2.115 | 12.780 | 13.671 | 76.622 | 6.667 | 14.250 | 66.59 |
| 124 | KY | 30.88 | 13.10 | 2.36 | 4.05 | 7.897 | 3.902 | 2.024 | 22.269 | 17.448 | 80.778 | 6.667 | 14.333 | 57.47 |
| 125 | KY | 22.17 | 11.33 | 1.96 | 3.24 | 6.675 | 3.033 | 2.206 | 14.181 | 14.736 | 76.579 | 6.333 | 13.667 | 69.42 |
| 126 | KY | 25.38 | 9.08 | 2.85 | 3.69 | 6.551 | 2.666 | 2.466 | 12.375 | 13.905 | 74.120 | 8.917 | 18.500 | 61.84 |
| 127 | KY | 23.71 | 12.29 | 1.95 | 3.43 | 7.751 | 2.936 | 2.644 | 16.135 | 16.633 | 71.552 | 6.667 | 14.250 | 53.28 |
| 128 | KY | 30.04 | 13.55 | 2.29 | 3.97 | 7.718 | 3.384 | 2.283 | 18.127 | 16.538 | 74.556 | 8.250 | 17.417 | 68.80 |
| 129 | KY | 26.14 | 11.54 | 2.28 | 3.45 | 7.044 | 2.823 | 2.514 | 14.746 | 15.133 | 76.664 | 7.167 | 15.333 | 57.96 |
| 130 | KY | 27.98 | 8.96 | 3.14 | 3.13 | 6.686 | 3.270 | 2.039 | 15.995 | 15.023 | 81.555 | 8.250 | 17.417 | 57.93 |
| 131 | KY | 26.13 | 11.58 | 2.27 | 3.45 | 6.697 | 3.301 | 2.025 | 16.094 | 14.843 | 81.460 | 6.500 | 14.000 | 67.48 |
| 132 | KY | 27.96 | 12.40 | 2.26 | 3.51 | 7.552 | 2.883 | 2.628 | 15.615 | 15.940 | 72.062 | 7.500 | 15.833 | 60.53 |
| 133 | KY | 25.03 | 11.08 | 2.29 | 3.32 | 6.638 | 2.939 | 2.256 | 14.208 | 14.853 | 78.684 | 6.750 | 14.500 | 63.62 |
| 134 | TN | 22.87 | 9.70 | 2.37 | 3.87 | 5.789 | 2.871 | 2.020 | 12.217 | 12.997 | 81.585 | 6.417 | 13.750 | 64.12 |
| 135 | TN | 26.43 | 9.78 | 2.71 | 3.53 | 5.577 | 2.861 | 1.948 | 11.823 | 12.815 | 83.765 | 7.500 | 16.000 | 64.67 |
| 136 | TN | 23.25 | 8.85 | 2.65 | 2.98 | 5.246 | 2.169 | 2.431 | 8.108 | 11.443 | 74.776 | 7.917 | 16.750 | 61.29 |
| 137 | TN | 25.08 | 10.80 | 2.36 | 3.38 | 7.073 | 3.066 | 2.303 | 15.676 | 16.338 | 77.346 | 6.500 | 13.917 | 77.49 |
| 138 | TN | 27.33 | 12.17 | 2.25 | 3.69 | 7.926 | 3.157 | 2.542 | 17.637 | 16.766 | 71.955 | 6.083 | 13.167 | 60.08 |
| 139 | TN | 21.08 | 8.21 | 2.59 | 2.73 | 4.887 | 2.837 | 1.726 | 9.629 | 13.200 | 81.807 | 6.417 | 13.833 | 63.57 |
| 140 | TN | 28.79 | 11.00 | 2.63 | 3.52 | 6.478 | 3.682 | 1.765 | 17.518 | 15.762 | 85.958 | 7.667 | 16.250 | 73.50 |
| 141 | TN | 38.51 | 14.65 | 2.65 | 4.99 | 8.833 | 3.728 | 2.372 | 24.153 | 19.164 | 77.374 | 8.083 | 17.167 | 60.86 |
| 142 | TN | 27.17 | 12.96 | 2.10 | 3.75 | 7.579 | 3.111 | 2.445 | 17.459 | 16.431 | 77.595 | 7.583 | 16.167 | 39.83 |
| 143 | TN | 24.83 | 11.65 | 2.14 | 3.90 | 6.943 | 2.835 | 2.448 | 13.736 | 14.664 | 72.798 | 6.583 | 14.167 | 40.33 |
| 144 | TN | 25.84 | 12.10 | 2.14 | 4.18 | 7.401 | 3.009 | 2.466 | 15.616 | 15.793 | 73.165 | 7.083 | 15.167 | 46.97 |
| 145 | TN | 25.76 | 10.30 | 2.51 | 4.18 | 6.263 | 2.438 | 2.575 | 10.848 | 13.281 | 72.485 | 7.167 | 15.333 | 43.05 |
| 146 | TN | 24.80 | 10.91 | 2.29 | 4.60 | 6.613 | 2.662 | 2.504 | 12.256 | 13.979 | 72.155 | 6.583 | 14.083 | 36.40 |
| 147 | TN | 29.79 | 10.33 | 2.90 | 3.81 | 5.986 | 2.624 | 2.280 | 11.039 | 13.354 | 75.341 | 7.083 | 15.667 | 79.27 |
| 148 | MS | 30.89 | 11.86 | 2.61 | 3.60 | 7.057 | 2.869 | 2.473 | 14.357 | 14.898 | 74.098 | 9.500 | 20.000 | 42.09 |
| 149 | MS | 30.63 | 8.06 | 3.88 | 3.13 | 6.222 | 2.224 | 2.798 | 9.764 | 13.110 | 69.255 | 11.083 | 23.000 | 48.84 |
| 150 | MS | 23.18 | 9.89 | 2.36 | 3.01 | 6.056 | 3.000 | 2.021 | 13.098 | 13.734 | 81.127 | 7.333 | 15.667 | 66.03 |
| 151 | MS | 26.75 | 9.61 | 2.82 | 3.59 | 5.676 | 2.901 | 1.951 | 11.769 | 13.817 | 80.410 | 7.167 | 15.250 | 65.46 |
| 152 | AL | 27.30 | 10.85 | 2.52 | 4.00 | 6.615 | 2.905 | 2.294 | 13.899 | 14.358 | 77.673 | 9.083 | 19.083 | 55.22 |
| 153 | AL | 26.20 | 11.34 | 2.33 | 4.03 | 7.079 | 3.442 | 2.065 | 17.092 | 15.424 | 78.102 | 6.250 | 13.500 | 57.82 |
| 154 | AL | 28.13 | 11.69 | 2.42 | 3.63 | 6.542 | 2.962 | 2.213 | 14.607 | 14.658 | 81.709 | 9.417 | 19.667 | 60.94 |
| 155 | AL | 28.12 | 10.40 | 2.71 | 2.88 | 6.870 | 3.038 | 2.272 | 15.338 | 15.468 | 78.724 | 9.083 | 19.167 | 63.74 |
| 156 | AL | 31.35 | 11.00 | 2.86 | 3.83 | 6.053 | 2.894 | 2.094 | 12.577 | 13.813 | 79.635 | 8.583 | 18.000 | 67.36 |
| 157 | AL | 32.80 | 12.44 | 2.66 | 4.60 | 7.665 | 3.391 | 2.267 | 18.328 | 17.563 | 76.151 | 9.083 | 19.083 | 71.08 |
| 158 | MS | 29.67 | 12.00 | 2.49 | 3.83 | 7.049 | 2.691 | 2.635 | 13.848 | 15.073 | 73.959 | 8.333 | 17.667 | 57.21 |
| 159 | MS | 26.12 | 10.93 | 2.39 | 3.56 | 6.373 | 2.789 | 2.290 | 12.651 | 13.913 | 76.298 | 9.250 | 19.333 | 70.64 |
| 160 | MS | 27.96 | 11.49 | 2.44 | 4.03 | 7.076 | 3.089 | 2.295 | 15.924 | 15.463 | 78.221 | 8.500 | 18.000 | 57.78 |
| 161 | MS | 29.00 | 12.29 | 2.37 | 3.85 | 7.407 | 2.824 | 2.631 | 15.179 | 15.912 | 73.672 | 8.333 | 17.667 | 51.33 |
| 162 | MS | 27.55 | 11.44 | 2.44 | 4.62 | 7.153 | 3.277 | 2.191 | 16.634 | 16.317 | 77.521 | 6.833 | 14.667 | 51.41 |
| 163 | MS | 27.63 | 12.90 | 2.16 | 4.06 | 7.382 | 3.063 | 2.405 | 15.652 | 16.128 | 72.572 | 7.750 | 16.500 | 59.34 |
| 164 | IA | 30.04 | 12.24 | 2.46 | 3.14 | 7.981 | 3.277 | 2.439 | 18.505 | 18.378 | 74.116 | 9.417 | 19.500 | 65.40 |
| 165 | IA | 28.58 | 10.76 | 2.67 | 3.24 | 6.928 | 2.881 | 2.425 | 14.803 | 15.105 | 78.189 | 9.417 | 19.667 | 62.71 |
| 166 | IA | 25.63 | 9.13 | 2.82 | 3.44 | 5.553 | 2.724 | 2.041 | 10.919 | 12.462 | 81.076 | 7.583 | 16.167 | 69.30 |
| 167 | IA | 26.91 | 9.85 | 2.76 | 3.33 | 6.913 | 2.678 | 2.590 | 13.510 | 15.025 | 74.381 | 8.833 | 18.667 | 47.18 |
| 168 | IA | 23.68 | 10.10 | 2.35 | 3.45 | 5.888 | 2.485 | 2.379 | 10.231 | 13.925 | 73.872 | 7.250 | 15.500 | 48.33 |
| 169 | MO | 31.83 | 13.58 | 2.35 | 4.43 | 8.368 | 3.373 | 2.483 | 20.185 | 18.925 | 74.270 | 8.000 | 17.000 | 53.38 |
| 170 | MO | 32.00 | 11.08 | 2.92 | 4.58 | 7.674 | 3.424 | 2.257 | 19.387 | 16.950 | 78.038 | 7.083 | 15.167 | 47.79 |
| 171 | MO | 27.14 | 13.15 | 2.07 | 3.56 | 6.920 | 2.900 | 2.396 | 14.987 | 15.268 | 77.762 | 9.333 | 19.583 | 51.45 |
| 172 | MO | 31.21 | 10.52 | 3.04 | 4.39 | 6.323 | 2.928 | 2.176 | 13.430 | 14.382 | 78.825 | 7.917 | 16.833 | 62.65 |
| 173 | MO | 26.09 | 10.88 | 2.51 | 3.21 | 6.728 | 2.763 | 2.449 | 13.255 | 14.247 | 74.598 | 8.500 | 18.000 | 44.17 |
| 174 | MO | 29.87 | 11.08 | 2.74 | 4.06 | 8.346 | 3.656 | 2.291 | 21.836 | 18.383 | 76.559 | 7.417 | 15.833 | 63.22 |
| 175 | MO | 24.74 | 11.20 | 2.23 | 4.43 | 6.923 | 3.168 | 2.192 | 15.962 | 15.488 | 79.354 | 6.500 | 14.000 | 51.60 |
| 176 | MO | 30.52 | 11.70 | 2.62 | 4.35 | 6.522 | 3.042 | 2.146 | 14.549 | 14.327 | 80.381 | 9.750 | 20.500 | 56.69 |
| 177 | MO | 24.37 | 9.03 | 2.70 | 3.73 | 5.701 | 2.761 | 2.077 | 11.377 | 12.764 | 80.503 | 8.167 | 17.167 | 71.29 |
| 178 | MO | 28.79 | 11.11 | 2.60 | 4.57 | 6.364 | 3.213 | 1.982 | 14.997 | 14.462 | 82.809 | 7.417 | 15.667 | 64.79 |
| 179 | MO | 27.49 | 10.92 | 2.53 | 3.74 | 6.390 | 3.255 | 1.966 | 15.787 | 14.913 | 85.699 | 8.167 | 17.333 | 47.98 |
| 180 | MO | 26.43 | 10.44 | 2.54 | 4.08 | 6.428 | 3.214 | 2.009 | 14.962 | 14.523 | 80.768 | 7.000 | 15.000 | 61.91 |
| 181 | KS | 26.92 | 9.83 | 2.74 | 3.38 | 5.878 | 2.139 | 2.757 | 9.112 | 12.542 | 71.449 | 9.833 | 20.667 | 52.11 |
| 182 | KS | 32.50 | 12.43 | 2.63 | 3.63 | 7.336 | 2.622 | 2.809 | 14.007 | 15.573 | 71.681 | 10.000 | 21.000 | 76.28 |
| 183 | OK | 27.03 | 10.98 | 2.53 | 3.56 | 6.970 | 2.668 | 2.618 | 13.236 | 14.988 | 72.314 | 7.917 | 16.750 | 53.97 |
| 184 | OK | 31.15 | 10.37 | 3.04 | 4.60 | 7.734 | 3.734 | 2.073 | 20.449 | 17.297 | 78.783 | 8.417 | 17.500 | 65.68 |
| 185 | OK | 31.71 | 12.79 | 2.49 | 4.48 | 7.440 | 3.322 | 2.287 | 17.547 | 16.382 | 75.956 | 8.417 | 17.833 | 62.26 |
| 186 | OK | 25.00 | 11.08 | 2.29 | 3.76 | 6.654 | 3.326 | 2.004 | 15.730 | 15.022 | 80.200 | 7.083 | 15.167 | 59.97 |
| 187 | OK | 31.79 | 11.17 | 2.84 | 3.89 | 6.477 | 3.185 | 2.034 | 14.712 | 14.475 | 79.931 | 8.667 | 18.333 | 70.65 |
| 188 | OK | 25.15 | 10.22 | 2.47 | 3.43 | 5.948 | 2.824 | 2.111 | 12.030 | 13.012 | 79.338 | 8.000 | 16.917 | 64.97 |
| 189 | AR | 24.51 | 10.90 | 2.26 | 3.28 | 6.673 | 3.031 | 2.214 | 14.149 | 15.808 | 75.749 | 7.167 | 15.333 | 54.13 |
| 190 | AR | 32.30 | 13.53 | 2.40 | 3.91 | 7.933 | 3.467 | 2.293 | 19.352 | 17.553 | 75.120 | 8.250 | 17.500 | 48.16 |
| 191 | AR | 28.04 | 10.38 | 2.73 | 3.43 | 7.423 | 2.998 | 2.497 | 16.257 | 16.163 | 75.913 | 8.167 | 17.167 | 69.24 |
| 192 | CN | 20.52 | 8.40 | 2.45 | 2.48 | 5.151 | 2.205 | 2.340 | 8.078 | 11.078 | 75.357 | 7.667 | 16.333 | 74.15 |
| 193 | CN | 27.79 | 10.79 | 2.60 | 3.83 | 6.111 | 3.053 | 2.008 | 13.320 | 13.968 | 78.872 | 6.167 | 13.333 | 75.57 |
| 194 | CN | 25.17 | 13.07 | 1.95 | 4.03 | 7.394 | 3.183 | 2.328 | 16.952 | 16.124 | 76.618 | 5.667 | 12.333 | 64.24 |
| 195 | CN | 23.18 | 9.32 | 2.53 | 3.68 | 5.824 | 3.228 | 1.818 | 13.661 | 13.585 | 83.964 | 7.417 | 15.667 | 63.49 |
| 196 | CN | 26.92 | 12.01 | 2.25 | 3.79 | 7.133 | 3.310 | 2.157 | 17.618 | 15.869 | 81.846 | 7.417 | 15.833 | 38.93 |
| 197 | CN | 24.15 | 9.83 | 2.47 | 3.30 | 5.962 | 2.848 | 2.089 | 12.226 | 12.955 | 79.961 | 6.417 | 13.833 | 69.01 |
| 198 | CN | 27.93 | 9.23 | 3.05 | 3.64 | 7.204 | 3.288 | 2.198 | 16.803 | 15.938 | 77.589 | 8.000 | 16.917 | 54.37 |
| 199 | CN | 31.81 | 12.54 | 2.56 | 5.02 | 7.518 | 3.442 | 2.190 | 18.404 | 16.268 | 77.642 | 8.250 | 17.500 | 39.47 |
| 200 | CN | 24.88 | 10.93 | 2.28 | 3.52 | 6.514 | 2.835 | 2.303 | 13.372 | 14.216 | 77.701 | 7.250 | 15.250 | 53.08 |
| 201 | CN | 29.59 | 13.80 | 2.15 | 3.68 | 7.809 | 3.482 | 2.245 | 18.712 | 19.078 | 74.490 | 7.083 | 15.333 | 62.70 |
| 202 | CN | 24.53 | 11.14 | 2.20 | 3.55 | 6.728 | 2.978 | 2.266 | 14.614 | 14.643 | 78.831 | 6.833 | 14.667 | 53.68 |
| 203 | CN | 26.85 | 11.95 | 2.27 | 3.13 | 7.083 | 3.235 | 2.190 | 15.789 | 15.375 | 75.285 | 8.250 | 17.500 | 49.71 |
| 204 | CN | 26.10 | 13.42 | 1.95 | 4.04 | 7.944 | 3.083 | 2.580 | 17.214 | 16.997 | 71.527 | 7.667 | 16.333 | 45.68 |
| 205 | CN | 22.81 | 10.86 | 2.11 | 3.04 | 6.731 | 2.485 | 2.712 | 11.772 | 14.225 | 70.018 | 6.583 | 14.167 | 45.73 |
| 206 | CN | 26.53 | 11.87 | 2.24 | 3.79 | 6.758 | 2.638 | 2.577 | 12.832 | 14.430 | 73.523 | 7.583 | 16.167 | 58.06 |
| 207 | CN | 23.49 | 9.90 | 2.40 | 3.55 | 5.763 | 2.590 | 2.224 | 10.801 | 12.663 | 78.560 | 6.833 | 14.667 | 95.95 |
| 208 | CN | 29.46 | 12.06 | 2.45 | 3.13 | 7.013 | 2.797 | 2.514 | 14.364 | 15.259 | 75.603 | 8.750 | 18.500 | 67.70 |
| 209 | CN | 27.38 | 12.28 | 2.23 | 3.28 | 6.480 | 2.991 | 2.166 | 13.743 | 14.398 | 77.534 | 6.667 | 14.333 | 60.96 |
| 210 | CN | 28.92 | 11.88 | 2.44 | 3.68 | 6.944 | 3.156 | 2.204 | 16.334 | 15.523 | 81.236 | 7.250 | 15.500 | 42.48 |
| 211 | CN | 24.69 | 10.87 | 2.28 | 3.31 | 6.397 | 3.165 | 2.025 | 14.558 | 14.318 | 81.049 | 6.917 | 14.833 | 71.05 |
| 212 | CN | 30.56 | 11.08 | 2.78 | 3.52 | 6.818 | 2.638 | 2.601 | 12.724 | 14.438 | 72.018 | 7.667 | 16.250 | 61.63 |
| 213 | CN | 30.25 | 12.21 | 2.48 | 4.23 | 7.387 | 3.086 | 2.403 | 16.274 | 15.907 | 75.502 | 8.083 | 17.167 | 53.97 |
| 214 | CN | 24.31 | 10.68 | 2.29 | 3.38 | 6.769 | 2.719 | 2.492 | 13.303 | 14.653 | 74.941 | 6.833 | 14.667 | 61.68 |

**Raw data of Traits**

**S6 Table. Physiological traits data of different provenances.**

| No. | Provenances | Chl | Spro | PRO |
| --- | --- | --- | --- | --- |
| 1 | VA | 32.89 | 1257.956 | 31.425 |
| 2 | VA | 34.60 | 786.958 | 27.185 |
| 3 | VA | 41.78 | 507.130 | 57.286 |
| 4 | VA | 42.03 | 360.053 | 24.838 |
| 5 | VA | 34.61 | 431.522 | 20.397 |
| 6 | VA | 36.87 | 552.176 | 17.062 |
| 7 | VA | 30.26 | 635.584 | 13.492 |
| 8 | VA | 34.64 | 1327.356 | 28.492 |
| 9 | VA | 32.78 | 1053.894 | 30.956 |
| 10 | VA | 38.88 | 450.941 | 105.102 |
| 11 | VA | 32.79 | 729.656 | 25.173 |
| 12 | VA | 31.23 | 1248.087 | 47.582 |
| 13 | VA | 38.59 | 1906.432 | 31.827 |
| 14 | VA | 33.28 | 755.124 | 23.213 |
| 15 | VA | 33.53 | 1472.523 | 51.771 |
| 16 | VA | 37.79 | 1169.614 | 20.364 |
| 17 | VA | 39.28 | 1067.105 | 33.771 |
| 18 | VA | 32.33 | 886.442 | 15.369 |
| 19 | VA | 33.04 | 1539.376 | 23.196 |
| 20 | WV | 32.84 | 322.010 | 13.123 |
| 21 | WV | 33.83 | 971.760 | 35.062 |
| 22 | WV | 37.84 | 1145.260 | 17.246 |
| 23 | WV | 36.14 | 665.986 | 17.430 |
| 24 | WV | 43.12 | 469.246 | 37.794 |
| 25 | WV | 31.28 | 694.160 | 23.564 |
| 26 | WV | 31.27 | 828.503 | 46.107 |
| 27 | WV | 36.14 | 1161.018 | 61.743 |
| 28 | WV | 32.51 | 1286.448 | 25.492 |
| 29 | WV | 39.12 | 427.065 | 26.668 |
| 30 | WV | 37.54 | 996.750 | 28.236 |
| 31 | WV | 39.87 | 663.598 | 14.245 |
| 32 | WV | 38.14 | 1472.364 | 33.570 |
| 33 | WV | 36.40 | 863.044 | 15.621 |
| 34 | WV | 35.60 | 1367.627 | 42.654 |
| 35 | WV | 43.53 | 910.955 | 35.616 |
| 36 | WV | 36.53 | 619.666 | 51.151 |
| 37 | WV | 41.24 | 634.788 | 35.011 |
| 38 | WV | 39.39 | 1834.008 | 89.749 |
| 39 | WV | 38.82 | 2088.050 | 166.629 |
| 40 | NC | 41.31 | 659.301 | 27.905 |
| 41 | NC | 32.68 | 790.619 | 20.967 |
| 42 | NC | 44.90 | 722.970 | 13.861 |
| 43 | NC | 36.73 | 689.703 | 20.061 |
| 44 | NC | 32.79 | 652.456 | 25.643 |
| 45 | NC | 31.13 | 832.323 | 23.816 |
| 46 | GA | 34.43 | 894.242 | 28.074 |
| 47 | GA | 31.38 | 1143.350 | 31.475 |
| 48 | GA | 29.43 | 1315.736 | 36.704 |
| 49 | GA | 30.71 | 507.130 | 15.855 |
| 50 | GA | 32.56 | 2281.129 | 23.447 |
| 51 | GA | 35.59 | 483.731 | 19.877 |
| 52 | GA | 33.30 | 814.177 | 26.866 |
| 53 | MD | 41.48 | 706.257 | 23.883 |
| 54 | MD | 34.98 | 653.729 | 33.771 |
| 55 | MD | 39.97 | 314.688 | 24.788 |
| 56 | MD | 34.22 | 747.961 | 22.559 |
| 57 | MD | 38.34 | 456.353 | 22.056 |
| 58 | PA | 35.32 | 1216.729 | 38.296 |
| 59 | PA | 33.42 | 607.091 | 31.241 |
| 60 | PA | 36.44 | 334.266 | 13.123 |
| 61 | PA | 40.39 | 483.413 | 30.905 |
| 62 | PA | 31.93 | 555.519 | 24.586 |
| 63 | PA | 36.49 | 598.337 | 30.620 |
| 64 | PA | 36.48 | 520.023 | 29.529 |
| 65 | PA | 35.64 | 669.329 | 18.822 |
| 66 | PA | 38.51 | 706.575 | 31.526 |
| 67 | PA | 38.04 | 869.411 | 29.665 |
| 68 | PA | 34.88 | 637.812 | 18.453 |
| 69 | PA | 38.34 | 555.041 | 16.324 |
| 70 | PA | 38.54 | 1817.454 | 18.101 |
| 71 | PA | 36.03 | 966.826 | 21.034 |
| 72 | OH | 43.17 | 1113.903 | 13.542 |
| 73 | OH | 36.20 | 762.923 | 20.046 |
| 74 | OH | 34.99 | 845.535 | 19.777 |
| 75 | OH | 33.04 | 634.947 | 84.738 |
| 76 | OH | 33.37 | 546.923 | 24.620 |
| 77 | OH | 36.50 | 592.447 | 78.855 |
| 78 | OH | 36.20 | 2187.216 | 132.235 |
| 79 | OH | 35.29 | 1031.610 | 22.274 |
| 80 | OH | 35.10 | 836.462 | 43.961 |
| 81 | OH | 38.58 | 1326.401 | 21.973 |
| 82 | OH | 33.71 | 1709.374 | 29.113 |
| 83 | IN | 36.64 | 675.218 | 37.877 |
| 84 | IN | 44.42 | 429.612 | 38.296 |
| 85 | IN | 41.78 | 594.039 | 19.358 |
| 86 | IN | 38.40 | 705.779 | 25.728 |
| 87 | IN | 36.14 | 882.781 | 33.197 |
| 88 | IN | 40.19 | 430.408 | 16.710 |
| 89 | IN | 39.89 | 1629.787 | 51.486 |
| 90 | IN | 39.69 | 1216.729 | 75.269 |
| 91 | IN | 37.44 | 1188.874 | 26.179 |
| 92 | IN | 33.91 | 1300.137 | 38.430 |
| 93 | IN | 40.97 | 1120.906 | 24.519 |
| 94 | IN | 35.60 | 2078.659 | 30.788 |
| 95 | IN | 37.53 | 1421.587 | 62.531 |
| 96 | IN | 37.13 | 1204.951 | 67.844 |
| 97 | IN | 35.64 | 597.700 | 45.788 |
| 98 | IN | 34.14 | 789.824 | 35.665 |
| 99 | IN | 40.13 | 2063.060 | 24.067 |
| 100 | IL | 33.23 | 370.240 | 37.812 |
| 101 | IL | 38.69 | 797.146 | 22.190 |
| 102 | IL | 36.03 | 779.000 | 21.603 |
| 103 | IL | 38.27 | 781.387 | 22.911 |
| 104 | IL | 38.84 | 1785.301 | 40.157 |
| 105 | IL | 37.80 | 662.166 | 30.991 |
| 106 | IL | 35.87 | 1417.289 | 36.302 |
| 107 | IL | 44.42 | 602.316 | 46.493 |
| 108 | IL | 40.70 | 899.176 | 47.849 |
| 109 | IL | 38.73 | 502.832 | 16.626 |
| 110 | IL | 41.02 | 1118.360 | 23.279 |
| 111 | IL | 34.57 | 852.697 | 23.900 |
| 112 | KY | 41.78 | 974.943 | 81.487 |
| 113 | KY | 36.72 | 2118.612 | 75.419 |
| 114 | KY | 33.49 | 632.559 | 30.386 |
| 115 | KY | 38.66 | 702.118 | 21.520 |
| 116 | KY | 34.59 | 776.612 | 20.129 |
| 117 | KY | 35.70 | 1472.045 | 23.078 |
| 118 | KY | 35.08 | 631.127 | 208.645 |
| 119 | KY | 35.10 | 754.964 | 15.553 |
| 120 | KY | 36.40 | 616.642 | 31.441 |
| 121 | KY | 37.26 | 797.941 | 16.172 |
| 122 | KY | 38.17 | 1096.075 | 21.905 |
| 123 | KY | 38.40 | 482.935 | 37.810 |
| 124 | KY | 36.38 | 883.577 | 20.229 |
| 125 | KY | 30.24 | 575.734 | 19.006 |
| 126 | KY | 32.84 | 1832.575 | 31.006 |
| 127 | KY | 29.84 | 1365.080 | 82.861 |
| 128 | KY | 35.99 | 779.955 | 24.638 |
| 129 | KY | 45.76 | 1046.890 | 23.598 |
| 130 | KY | 35.06 | 915.731 | 60.973 |
| 131 | KY | 35.02 | 1809.336 | 29.933 |
| 132 | KY | 30.63 | 1399.780 | 22.861 |
| 133 | KY | 38.58 | 669.806 | 16.659 |
| 134 | TN | 39.74 | 915.890 | 29.749 |
| 135 | TN | 34.58 | 582.419 | 26.615 |
| 136 | TN | 28.70 | 673.467 | 26.515 |
| 137 | TN | 32.11 | 510.472 | 13.827 |
| 138 | TN | 32.88 | 509.995 | 18.620 |
| 139 | TN | 35.64 | 1265.596 | 21.486 |
| 140 | TN | 31.60 | 175.729 | 27.084 |
| 141 | TN | 32.00 | 1005.664 | 14.279 |
| 142 | TN | 37.80 | 1176.617 | 25.509 |
| 143 | TN | 34.37 | 293.358 | 42.151 |
| 144 | TN | 35.47 | 627.625 | 33.570 |
| 145 | TN | 35.53 | 804.468 | 35.983 |
| 146 | TN | 32.92 | 937.537 | 33.621 |
| 147 | TN | 32.20 | 954.410 | 23.849 |
| 148 | MS | 37.61 | 1121.384 | 21.537 |
| 149 | MS | 38.10 | 917.959 | 16.643 |
| 150 | MS | 30.01 | 915.412 | 23.062 |
| 151 | MS | 30.70 | 795.713 | 23.414 |
| 152 | AL | 36.98 | 1507.859 | 41.816 |
| 153 | AL | 35.97 | 671.239 | 39.051 |
| 154 | AL | 36.03 | 1002.799 | 38.364 |
| 155 | AL | 37.64 | 488.347 | 18.872 |
| 156 | AL | 40.70 | 670.284 | 24.838 |
| 157 | AL | 32.58 | 566.979 | 13.693 |
| 158 | MS | 37.86 | 341.748 | 27.670 |
| 159 | MS | 37.27 | 820.385 | 18.738 |
| 160 | MS | 37.59 | 925.122 | 30.168 |
| 161 | MS | 34.01 | 617.438 | 41.414 |
| 162 | MS | 33.62 | 821.499 | 15.134 |
| 163 | MS | 31.18 | 597.859 | 23.179 |
| 164 | IA | 39.89 | 472.748 | 47.245 |
| 165 | IA | 36.81 | 792.370 | 20.665 |
| 166 | IA | 32.42 | 459.377 | 49.558 |
| 167 | IA | 39.97 | 740.002 | 67.441 |
| 168 | IA | 38.66 | 403.189 | 25.858 |
| 169 | MO | 36.02 | 592.288 | 36.637 |
| 170 | MO | 34.98 | 459.218 | 18.973 |
| 171 | MO | 41.62 | 1133.163 | 35.732 |
| 172 | MO | 34.12 | 392.047 | 40.007 |
| 173 | MO | 38.40 | 1170.728 | 24.872 |
| 174 | MO | 35.37 | 695.115 | 40.964 |
| 175 | MO | 35.70 | 481.184 | 21.118 |
| 176 | MO | 34.84 | 993.249 | 29.347 |
| 177 | MO | 37.07 | 658.186 | 56.197 |
| 178 | MO | 41.03 | 985.767 | 28.693 |
| 179 | MO | 36.70 | 573.824 | 13.224 |
| 180 | MO | 33.53 | 603.430 | 25.257 |
| 181 | KS | 32.51 | 545.968 | 32.246 |
| 182 | KS | 31.44 | 2345.754 | 29.883 |
| 183 | OK | 34.79 | 949.953 | 15.972 |
| 184 | OK | 36.54 | 640.040 | 11.414 |
| 185 | OK | 33.01 | 737.933 | 10.861 |
| 186 | OK | 32.19 | 1152.264 | 19.375 |
| 187 | OK | 26.09 | 569.049 | 33.602 |
| 188 | OK | 32.37 | 926.077 | 24.318 |
| 189 | AR | 35.97 | 873.709 | 26.464 |
| 190 | AR | 34.47 | 510.632 | 15.000 |
| 191 | AR | 33.68 | 296.224 | 19.408 |
| 192 | CN | 28.51 | 1819.046 | 84.973 |
| 193 | CN | 36.14 | 527.663 | 28.693 |
| 194 | CN | 33.83 | 594.357 | 26.715 |
| 195 | CN | 33.53 | 2424.704 | 31.542 |
| 196 | CN | 36.63 | 896.152 | 23.380 |
| 197 | CN | 34.81 | 876.892 | 26.682 |
| 198 | CN | 37.63 | 954.569 | 24.772 |
| 199 | CN | 36.17 | 1760.788 | 23.495 |
| 200 | CN | 39.53 | 319.622 | 11.113 |
| 201 | CN | 36.21 | 1040.842 | 29.499 |
| 202 | CN | 42.50 | 895.515 | 25.861 |
| 203 | CN | 38.60 | 1079.203 | 49.542 |
| 204 | CN | 34.19 | 1161.973 | 24.603 |
| 205 | CN | 37.16 | 1066.469 | 29.749 |
| 206 | CN | 31.81 | 516.044 | 19.205 |
| 207 | CN | 37.29 | 1013.304 | 22.157 |
| 208 | CN | 36.08 | 980.833 | 30.402 |
| 209 | CN | 33.26 | 745.255 | 25.559 |
| 210 | CN | 37.73 | 926.873 | 29.485 |
| 211 | CN | 35.80 | 656.276 | 19.173 |
| 212 | CN | 38.88 | 789.346 | 20.448 |
| 213 | CN | 38.12 | 955.524 | 23.564 |
| 214 | CN | 31.60 | 509.040 | 19.006 |
